# Risk-modeling of dog osteosarcoma genome scans shows individuals with Mendelian-level polygenic risk are common

**DOI:** 10.1186/s12864-019-5531-6

**Published:** 2019-03-19

**Authors:** Isain Zapata, Luis E. Moraes, Elise M. Fiala, Sara Zaldivar-Lopez, C. Guillermo Couto, Jennie L. Rowell, Carlos E. Alvarez

**Affiliations:** 1 Department of Veterinary Clinical Sciences, The Ohio State University College of Veterinary Medicine, Columbus, OH USA; 2 Department of Animal Sciences, The Ohio State University College of Food, Agricultural and Environmental Sciences, Columbus, OH USA; 3 Center for Molecular and Human Genetics, The Research Institute at Nationwide Children’s Hospital, Columbus, OH USA; 4 Present address: Clinical Genetics Service, Memorial Sloan Kettering Cancer Center, New York, NY USA; 5 Present address: Genomics and Animal Breeding Group, Department of Genetics, Faculty of Veterinary Medicine, University of Cordoba, 14071 Córdoba, Spain; 6 Couto Veterinary Consultants, Hilliard, OH USA; 7 Department of Nursing, The Ohio State University College of Nursing, Columbus, OH USA; 8 Department of Pediatrics, The Ohio State University College of Medicine, Columbus, OH USA

**Keywords:** Osteosarcoma, Canine, Breed, Intersection union test, Stepwise, Logistic regression modeling, LASSO, FGF9, EWSR1, Retrogene, TANGO2, OTX2, MTMR7, MTMR9, MARCO, BMPER, FBRSL1, NELL1, BRINP3, AQP4, CDKN2A, CDKN2B, IGF1, MIR100HG, SVIL

## Abstract

**Background:**

Despite the tremendous therapeutic advances that have stemmed from somatic oncogenetics, survival of some cancers has not improved in 50 years. Osteosarcoma still has a 5-year survival rate of 66%. We propose the natural canine osteosarcoma model can change that: it is extremely similar to the human condition, except for being highly heritable and having a dramatically higher incidence. Here we reanalyze published genome scans of osteosarcoma in three frequently-affected dog breeds and report entirely new understandings with immediate translational indications.

**Results:**

First, meta-analysis revealed association near *FGF9*, which has strong biological and therapeutic relevance. Secondly, risk-modeling by multiple logistic regression shows 22 of the 34 associated loci contribute to risk and eight have large effect sizes. We validated the Greyhound stepwise model in our own, independent, case-control cohort. Lastly, we updated the gene annotation from approximately 50 genes to 175, and prioritized those using cross-species genomics data. Mostly positional evidence suggests 13 genes are likely to be associated with mapped risk (including *MTMR9*, *EWSR1* retrogene, *TANGO2* and *FGF9*). Previous annotation included seven of those 13 and prioritized four by pathway enrichment. Ten of our 13 priority genes are in loci that contribute to risk modeling and thus can be studied epidemiologically and translationally in pet dogs. Other new candidates include *MYCN*, *SVIL* and *MIR100HG*.

**Conclusions:**

Polygenic osteosarcoma-risk commonly rises to Mendelian-levels in some dog breeds. This justifies caninized animal models and targeted clinical trials in pet dogs (e.g., using CDK4/6 and FGFR1/2 inhibitors).

**Electronic supplementary material:**

The online version of this article (10.1186/s12864-019-5531-6) contains supplementary material, which is available to authorized users.

## Background

Osteosarcoma is the most common pediatric and adult cancer of the bone in humans and dogs [1–3]. Surgery alone is rarely curative, but chemotherapy combinations initiated in the 1970’s have increased five-year survival to the current rate of 66% [1]. The inability to improve survival in the last 50 years shows a great unmet need for targeted therapies. Human and dog osteosarcoma are very similar by essentially all known measures – including epidemiology, genetics, pathology and treatment [2, 4, 5]; where they differ is in a dramatically higher incidence in dogs (see below, this section). Most of the top somatic mutations associated with osteosarcoma are shared by humans and dogs, including *TP53, MYC, CDKN2A/B, PTEN, RUNX2* and *DLG2* [2, 3, 6–8]. Top-frequency genes that don’t overlap include *RB1* in humans, and *KIT* and *MDM2* in dogs. Thus, the osteoblast cell lineage (*RUNX2*), TP53 (*CDKN2B* and *MDM2*) and linked PI3K/AKT and RB/E2F pathways (the other 7 genes) are central in both species.

Animal models of human disease are necessary for dissection of pathological mechanisms and development of therapies [9]. Mice are ideal for genetic or drug screening, and for dissection and targeting of precise mechanisms. However, we think of mouse and dog models not as alternatives to each other, but as complementary. To a large extent, they represent two sides of the spectrum between cost and tractability vs. natural disease relevance. Aside from experimental studies, dog models [10] are similar to human biomedical subjects, but without most of the ethical and legal limitations [4, 9]. Each dog breed is on the order of 100-fold simpler than the dog or human population. In contrast to mice, dogs are outbred and tend to live in human environments, receive health care and live to old age.

Many advantages stem from natural cancer models, including epidemiology, the full spectrum of cancer development through metastasis, and treatment outcomes. For several cancers, such as lymphoma and osteosarcoma, the canine incidence is much higher than in humans. Most breeds have increased or decreased risk of one or more cancer types. One unique potential for dog breed models to contribute to human cancer knowledge is their vastly increased feasibility for mapping germ line risk [9]. In humans, it is generally difficult to map most of the variance explaining traits of complex genetics. Similarly, those advantages in comparison to humans can vastly increase the power of studies of epigenetics, integrative genomics, pleiotropy, epistasis, gene-environment interactions, etc. Moreover, genome wide association studies (GWAS’s) of diverse cancers have shown that humans have essentially no common variants of large effect sizes [9]. Three osteosarcoma GWAS’s have been conducted on European populations, yielding candidate genes in *GRM4* (OR = 1.57), lincRNA *AC017053.1* (OR = 1.39), *NFIB* (OR = 2.43) and, for survival in Europeans and Brazilians, *GLDC* (hazards ratio of 1.76) [11, 12].

Because dogs are bred by humans, even pathological variants of large effect can elude negative selection when they are associated with preferred traits [13]. However, prior to this study there was no evidence that germ line cancer risk-variations that are common across dog breeds have sufficient effect sizes to be clinically actionable [9]. Osteosarcoma incidence is 1.02/100,000 in humans and at least 13.9/100,000 for the full dog population [2, 5]. However, canine osteosarcoma is strongly associated with breeds of large body size [14]. Although canine osteosarcoma risk increases with age, small dog breeds that have 50% longer lifespans than large breeds have incidence rates close to zero. It is therefore important to be more precise about dog osteosarcoma risk (see Additional file 1: Text). Using weight as a proxy for size, essentially all increased risk pertains to dog breeds with > 23 kg standard weight – which is half the total dog population. The mean weight of this group is 34 kg, which correlates with an odds ratio (OR) of ~ 6–10; however, the group of dog breeds > 44 kg has an OR of 23. These large effects illustrate how germ line cancer genetics is vastly more tractable in dogs. By contrast, human osteosarcoma risk is challenging to understand due to low disease prevalence, low penetrance of associated variants, and socioeconomic factors (Additional file 1: Text).

The term “clinically actionable” can refer to anything that contributes to observation, diagnosis and treatment of patients. There are three main classes of actions instructed by knowledge of inherited genetic risk: therapeutic intervention, disease screening (e.g., initiation and interpretation) and life planning [15]. Somatic mutation profiles in tumors can be used for stratification and treatment design, and germ line risk variation of sufficiently large effect includes such utility. The norms for additive effect sizes in diseases of complex genetics (aka, polygenic risk scores) are the same as for Mendelian pathological variants [16]: regarded as small risk if the OR is between 1.0–1.5, moderate if > 1.5 and intermediate if > 3 (assuming the 95% confidence intervals do not include 1.0) [15]. High risk is relatively extremely-rare in humans and not defined. We consider an OR > 9 to be high risk, whereas formal guidelines consider the human APO E4 homozygous OR of 13 to be very high [16].

Clinical and direct-to-consumer genetic testing can motivate individuals to take both clinical and non-clinical actions. However, when variation carries low relative risk and has little predictive power, it is unclear what if any action is meaningful. Almost all known human risk alleles from complex trait GWAS’s fall into this category and have been recommended to be reported as risk alleles rather than pathological variants [16]. Polygenic risk scoring in humans can be powerful for various types of discovery such as pleiotropy or “phenome” mapping, molecular phenotyping and gene-environment interactions. However, it is of little use at the level of individuals and currently only explains 1–15% of the variation that distinguishes, say, high vs. low risk groups [17]. A related issue is that the statistical evidence of risk associations in GWAS’s is specific to those studies’ populations. This is particularly important in canine disease genetics, for which many Mendelian disease haplotypes are known but are frequently only present in one or a few breeds. There is thus a great need to better understand genetic risk in human and veterinary medicine, including additive effects in complex disease [15–17]. Here we estimate genetic risk of dog osteosarcoma within three breeds and in generalized models.

The landmark study of Karlsson, Lindblad-Toh and associates included three osteosarcoma GWAS’s in different breeds with high risk – Greyhound, Rottweiler and Irish Wolfhound – as well as supporting evidence for a *CDKN2A/B/ANRIL* haplotype in other breeds [18]. That locus was fine mapped and the peak GWA marker was nominated to be the functional variant through loss of a PAX5 binding site. Karlsson et al. reported 33 osteosarcoma risk loci that explained 55–85% of phenotype variance in each breed. While that level of variance-explained is astronomical compared to human complex traits, none of the risk loci exceeded an OR of 1.75. Given the high prevalence of osteosarcoma in dogs, the latter seemed surprising but it was suggested by the authors that this was likely explained by additional loci that are fixed in each of the breeds (i.e., that could not be mapped). That was supported by their finding that the top effect-size candidate in Greyhounds is fixed in the two other breeds and seven others are fixed in one other breed (we found that one of the latter is almost completely fixed in two breeds).

Here we used Karlsson et al.’s rich data [18] to generate deep new knowledge that is actionable. Our interest was not to reanalyze the osteosarcoma GWAS’s in individual breeds. Rather we extend the original studies to include a type of meta-analysis of the three breeds together, develop risk models that consider all mapped loci, validate those models in our Greyhound osteosarcoma case-control cohort, and update and expand the annotation of GWA loci. Our overarching goal is to better define the utility of this model for development of new therapies for dogs and humans, and for testing those in clinical studies in pet dogs. Our findings show the dog osteosarcoma model is more powerful than previously suggested, and indicate how it could immediately accelerate medical translation.

## Results

### Interbreed meta-analysis

We conducted a meta-analysis of Karlsson, Lindblad-Toh et al.’s osteosarcoma GWAS’s in three breeds [18] by adding an Intersection Union Test (IUT) to the individual GWAS’s. The IUT is well established and is considered the gold standard for some applications, such as bioequivalence [19]. Its use is growing in diverse types of genome wide genetics, including for identification of pleiotropy across GWAS’s [20–22]. Here, the IUT combines single-marker statistical tests of the three GWAS’s into an omnibus test that generates an overall *p*-value. In other words, the IUT tests for loci that are consistently shared across the three GWAS’s, but may not have strong enough effects for detection in any individual GWAS. Using this approach, we evaluated each pairwise breed combination and all three breeds combined. Since these tests result in two and three independent *p*-values, respectively, we calculated the Fisher’s combined probability to consolidate the multiple *p*-values into one (all *p*-values are given in Additional file 2: Table S1)*.* We set significance thresholds at a Bonferroni adjusted level, which was calculated on the total number of valid IUT tests. These Bonferroni threshold values ranged from 4.53 × 10^− 7^ to 5.80 × 10^− 7^.

**Fig. 1:**
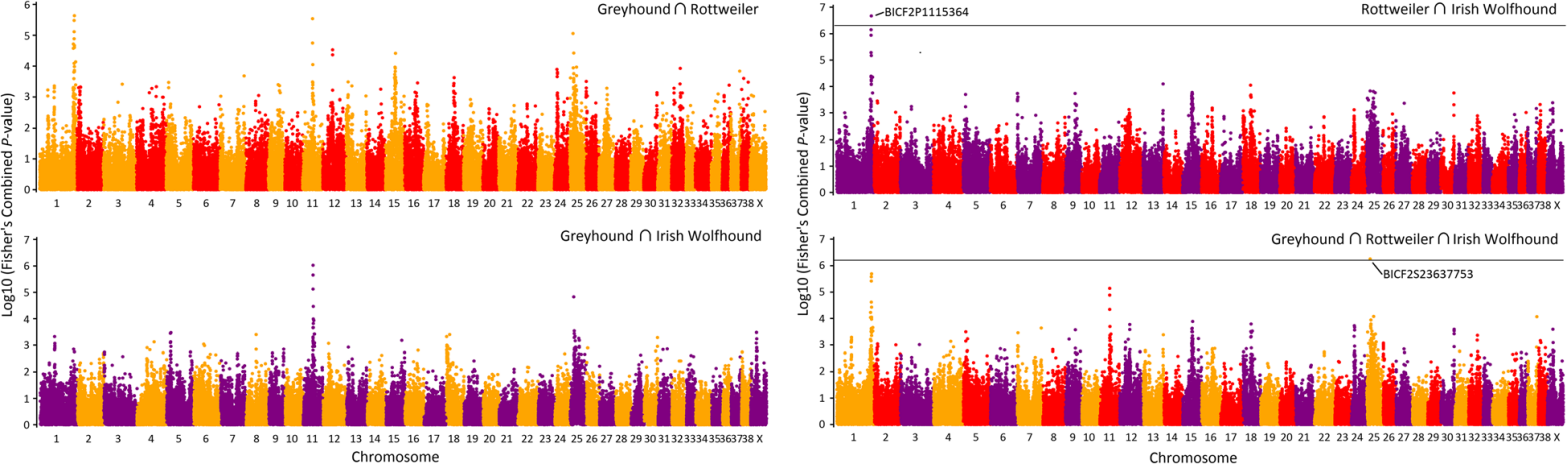
Manhattan plots of Intersect Union Test-Genome Wide Association Studies (IUT-GWAS) of dog Osteosarcoma in three breeds. Greyhound ∩ Rottweiler (top left), Greyhound ∩ Irish Wolfhound (bottom left), Rottweiler ∩ Irish Wolfhound (top right), and Greyhound ∩ Rottweiler ∩ Irish Wolfhound (bottom right). IUT-GWAS sets where significant hits were detected using a Bonferroni adjusted threshold have a horizontal line delimiting that threshold. Colors in the plot represent the breed included in the set represented: Greyhound, orange; Irish Wolfhound, purple and Rottweiler, red

In the Rottweiler-Irish Wolfhound set, we mapped a SNP at the 113,616,670 bp position in chromosome 1 (BICF2P1115364; canFam3.1 genome coordinates) that was originally reported as the top GWAS hit in the Rottweiler (Fig. 1). As this marker was not mapped in the Wolfhound, this finding validates the IUT method. In the three-breed set, we detected a previously unreported SNP at chr25:16,672,073 bp (BICF2523637753). Because no flanking markers are detected, the functional variant is likely to be old, to not be under selection, and to be located nearby. The SNP is 6.5 kb from the 5′-end of *FGF9* and there is no other gene in the vicinity. Our analyses of haplotype phasing and of selection showed the mapped haplotype spans *FGF9* and lacks signal of selection, respectively (Methods and Additional file 1: Text).

### Breed-specific and general models of osteosarcoma risk

A major interest in creating the models was to calculate the effect size of each locus included in them. None of the conditional OR values in the original work were above 1.75 (using EMMAX). We used all 33 originally reported risk loci plus the new *FGF9* locus to create breed-specific and generalized risk-models. We fitted multiple logistic regressions by using algorithms for two automated model selection methods: stepwise forwardoperator or LASSO (Table 1). Both have been used extensively to develop predictive models for clinical applications in cancer. As fewer variables are generally desirable for clinical and breeding purposes, we favored such models rather those that explain the most phenotype variance. As expected, that resulted in lower R^2^ values than originally reported: Greyhound, 54.2% vs. 56.9%; Wolfhound, 37.5% vs. 53.1%; and Rottweiler, 74.1% vs. 85.3%. The stepwise forward selection method generally performed well and had fewer variables. The exception was the Irish Wolfhound model, which retained all predictors. Unlike the original mapping results, some models shared markers across breeds.

**Table 1:** Multiple Logistic Regression model selection summary

	Cohort	Parameters	Max-rescaled R^2^	AIC (No Covariates)	AIC (With Covariates)	Terms in model	Hosmer & Lameshow Goodness of fit test
Stepwise forward selection	Generalized	14	0.377	645.95	538.75	BICF2P1225386, BICF2S23516022, BICF2P1194727, BICF2P1090686, BICF2P133066, BICF2S23118341, BICF2P66597, TIGRP2P200071, BICF2G630813090, BICF2S23533459, BICF2G63051809, BICF2G63095567, BICF2S23712115, BICF2P92014	0.209
	Greyhound	10	0.542	310.90	228.25	BICF2P1194727, BICF2P1421479, BICF2P133066, BICF2S23118341, BICF2P66597, BICF2G630418573, BICF2G63051809, BICF2G63095567, TIGRP2P331221, BICF2S23712115	0.8352
	Irish Wolfhound	4	0.375	173.18	147.74	BICF2P1225386, BICF2P1466354, BICF2G630590368, BICF2P1129874	0.4206
	Rottweiler	6	0.741	153.99	85.29	BICF2P1115364, BICF2P341331, BICF2G63095567, BICF2S23712115, TIGRP2P407733, BICF2P92014	0.6255
LASSO	Generalized	36	0.576	491.61	425.69	All terms were included by the method including cohort and sex	0.0217
	Greyhound	17	0.632	270.80	205.21	BICF2S23516022, TIGRP2P45171, BICF2P1194727, BICF2P1090686, BICF2P1421479, BICF2P133066, BICF2S23118341, BICF2P66597, BICF2G630418573, TIGRP2P215623, BICF2G630813090, BICF2G63051809, BICF2S23637753, BICF2S23325120, BICF2G63095567, TIGRP2P331221, BICF2S23712115	0.1729
	Irish Wolfhound	3	0.242	177.86	165.00	BICF2P1225386, BICF2S23746532, BICF2P1466354	0.0567
	Rottweiler	12	0.900	128.90	70.08	BICF2P1164085, BICF2P1115364, BICF2P1210630, BICF2P411325, BICF2P341331, TIGRP2P200071, BICF2S23533459, TIGRP2P286750, BICF2G63095567, BICF2S23712115, TIGRP2P407733, BICF2P92014	0.5324

Stepwise models showed no evidence of lack of fit. Overall the LASSO selection method was less successful. Although the R^2^ values were higher than originally reported for two breeds (Greyhounds, 63.2% vs. 56.9%; Wolfhound: 24.2% vs. 53.1%; and Rottweiler, 90.0% vs. 85.3%), the number of parameters included in the LASSO models was usually larger than in the stepwise models. Lack of fit tests for the LASSO models failed for the general and almost failed for the Wolfhound. This indicates those models are unsuitable for describing the data. Generalized models using both methods did not achieve the performance or simplicity of the breed models: stepwise had an R^2^ of 37.7% with 14 terms and LASSO had an R^2^ 57.6% with all terms.

Here we calculated OR estimates on a multiple logistic regression model with no intercept and no interaction terms. Heterozygous alleles were also included as a level for each class effect. We used Firth’s method since it allows for more precise estimations without the constraints of computationally-intensive exact logistic regression methods. In Additional file 3: Table S2 we present all OR estimates calculated by Firth’s logistic regression on all models selected by the two methods. It is important to note that OR estimates are calculated by estimating level combinations of the corresponding predictor marker while holding the other predictor variables constant at a certain reference value. ORs should be interpreted together with 95% confidence intervals, which serve as a measure of uncertainty. In our present analysis, we often observed cases of quasi-complete separation of the sample points, associated with large ORs and confidence intervals. This suggests that those effects are large, but that it is not possible to estimate reliable numeric values from the present sample. Our ORs are thus likely to be near the minimal-effect probability and the real effect could be larger.

Focusing on the stepwise forward selection models, the largest estimable effect for Greyhounds was at BICF2P13306 (chr11:41 Mb): OR of 7.47 for homozygotes and 2.92 for heterozygotes. In the original study, this marker had the lowest *p*-value in that breed and an OR of 1.26 (zygosity unspecified; the highest OR in this breed was 1.36). Nine other SNPs also had moderate to large effects or quasi-complete separation. All but two of the markers selected in the Greyhound model were mapped in the original study for the same breed. The largest estimable effect for Irish Wolfhound was for BICF2P1466354 (chr18:1 Mb): OR of 6.31. Markers BICF2G630590368 (chr32:22 Mb) and BICF2P1129874 (chr38:8 Mb) are notable because they were not mapped in this breed. The largest estimable effect for Rottweiler was an OR of 13.51 for BICF2P92014 (chr36:26 Mb).

### Validation of greyhound osteosarcoma risk models in a new cohort and risk inference in other breeds

We used our own Greyhound cohort of osteosarcoma cases and controls genotyped on the same platform for this study. We fitted the same stepwise forward and LASSO Greyhound models to assess their performance (Table 2). Correlation values were very close to those obtained at the model selection step. The lack of fit test was close to failing in the LASSO model but non-significantly. As expected, the model with fewer terms (stepwise forward selection) had a lower percent of correct calls. We consider a 90% correct rate to be satisfactory validation. In the Additional file 1: Text we present cluster analysis of breeds according to osteosarcoma risk loci (Additional file 4: Figure S1) and discuss risk inference in other breeds.

**Table 2:** Assessment of the Greyhound validation cohort with models from two selection methods

Method	Terms in model	Max-rescaled R^2^	Hosmer & Lameshow Goodness of fit test	% Correct calls
Stepwise forward selection	BICF2P1194727, BICF2P1421479, BICF2P133066, BICF2S23118341, BICF2P66597, BICF2G630418573, BICF2G63051809, BICF2G63095567, TIGRP2P331221, BICF2S23712115	0.5595	0.5390	91.0
LASSO	BICF2S23516022, TIGRP2P45171, BICF2P1194727, BICF2P1090686, BICF2P1421479, BICF2P133066, BICF2S23118341, BICF2P66597, BICF2G630418573, TIGRP2P215623, BICF2G630813090, BICF2G63051809, BICF2S23637753, BICF2S23325120, BICF2G63095567, TIGRP2P331221, BICF2S23712115	0.5921	0.0595	100

### Annotation and biological relevance of GWAS loci

**Fig. 2:**
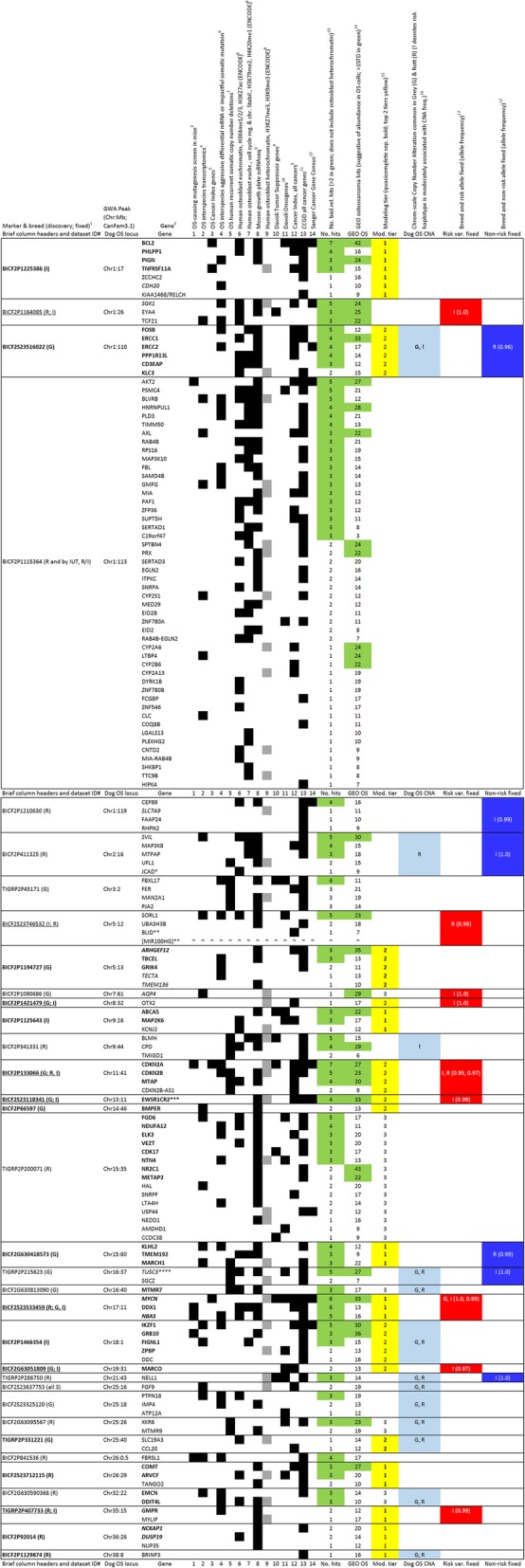
Genomics analysis of canine GWA mapped loci. All mapped intervals and up to 250 kb on each side were considered for genomics evidence of osteosarcoma, osteoblast or cancer genes. Only candidates with at least one hit are shown ^1^All loci were mapped by Karlsson et al. 2013 (PMID: 24330828) except one (FGF9 in this study); the BICF2S23637753 locus was mapped in this work using the same published data. In parentheses, the first breed is the discovery GWA breed (Karlsson et al. 2013, PMID: 24330828); the second or third are breeds fixed for the risk allele (derived in this work from Karlsson et al. 2013, PMID: 24330828, except Chr11:41 reported in that study). ^2^All genes with at least one hit for biological relevance categories here are shown. Genes most implicated by biological relevance and modeling are bold; genes that are not positionally top candidates at a locus are italicized. *JCAD is the official HUGO gene symbol, but most genomic data refers to it as KIAA1462; **refers to a miRNA cluster important in human cancer but is not represented in the biological relevance data types considered here; ***refers to data for the Ewing sarcoma gene EWSR1, the parent gene for canid-specific retrogene EWSR1CR; ****at 700 kb away, TUSC3 is the only gene beyond 200 kb outside the published interval, but is included here because it is an candidate OS tumor suppressor in dogs (PMID: 21837709). ^3^Transposon-based forward genetic screen for osteosarcoma development and metastasis (413 genes; Moriarity et al. 2015, PMID: 25961939) ^4^mRNA-sequening based osteosarcoma expression studies in humans, mice and dogs (Scott et al. 2018, PMID: 29066513) ^5^Cancer Index is a curated dataset of cancer types and associated germ line or somatic variant genes based on diverse types of evidence (contains 2168 cancer genes and 48 OS genes; Cotterill S.J. 2015, Cancer Genetics Web: http://www.cancer-genetics.org/) ^6^Transcriptomics of aggressive osteosarcomas in humans, dogs and mice (used genes with *p* < 0.05, FDR =/< 0.05: 3500 murine and 492 canine; Davis et al. 2017, PMID: 29100308) ^7^Human recurrent somatic copy number alterations in osteosarcoma (only observed overlap was in deleted genes) (Perry et al. 2014, PMID: 25512523) ^8^The integrated encyclopedia of DNA elements in the human genome, ENCODE Project Consortium, 2012 (PMID: 22955616) ^9^Single-cell RNA-sequencing analysis of the murine growth plate for bone elongation and regeneration (9739 genes; Li et al. 2016; PMID: 27160914) ^10^Statistical analysis of > 8200 human tumor-normal pairs in diverse cancer types to identify likely cancer driver properties (used top 1524 oncogenes, 1071 tumor suppressors; Davoli et al. 2013, PMID: 24183448) ^11^The Candidate Cancer Gene Database is a database of cancer driver genes from forward genetic screens in mice (9485 genes; Abbott et al. 2015, PMID: 25190456) ^12^Cancer Gene Census is a very stringent catalog of genes which contain mutations that have been causally implicated in cancer (719 genes; Futreal et al. 2004, PMID: 14993899) ^13^The number of hits for biological relevance categories marked by black boxes, but not heterochromatin (in grey) ^14^Number of hits searching each gene in the full Gene Expression Omnibus (Edgar et al. 2002, PMID: 11752295) for that term as a gene symbol together with the term osteosarcoma ^15^Modeling data from this work were arbitrarily classified into tier 1 (Odds Risk, OR > 9), 2 (OR 3–7.5) and 3 (OR 1.1–2.9) ^16^Comparative Genomic Hybridization (CGH) copy number alteration analysis by Thomas and Breen (Karlsson et al. 2013, PMID: 24330828) ^17^Allele frequencies of fixed or nearly-fixed risk and non-risk peak alleles were derived in this work using the Karlsson et al. 2013 genotype data (PMID: 24330828)

We updated the annotation for all 34 loci (Fig. 2 and Additional file 5: Table S3). The starting point was to use the updated dog genome assembly (canFam3) and annotation [23], and the human genome annotation to confidently identify all protein coding genes in the mapped intervals. We also checked gene content up to 250 kb on each side. [*TUSC3*, which is 700 kb outside the chr16:37 interval, was noticed because it is frequently deleted in canine osteosarcoma [24].] Canine non-coding RNA annotation is very limited so we focused on protein-coding genes. Analyses of selection and breed-specific phasing of risk-haplotypes are discussed in Additional file 1: Text (Additional file 6: Data and Additional file 5: Table S3).

Consideration of positional evidence together with biological relevance is complicated. Genetic mapping implicates haplotypes with wide ranges of gene densities, numbers of functional variants, molecular mechanisms and evolutionary histories. Marker position can be associated with enhancers that regulate distant genes or with elements that regulate epigenetic states. It is also unknown what fractions of osteosarcoma risk and development result from the cancer cell lineage vs other cells and tissues. Body size and sex hormones are associated with human and canine osteosarcoma [2, 14, 25]. Many tissues, including the hypothalamus and multiple cell types in the bone growth plate are involved in body size and involve sex hormone signaling.

Here we use data mining of resources that did not exist when Karlsson et al. conducted their osteosarcoma mapping in dogs [18]. Figure 2 lists the candidate genes with at least one occurrence in an analysis of genomic data for osteosarcoma, bone development and cancer in humans, mice and dogs (the full annotation of all loci is provided in Additional file 5: Table S3). These data are likely to be predominantly noise and are not intended to give mechanistic understanding. Rather, this information is meant to identify, rank and facilitate the consideration of possible candidates. In the Additional file 1: Text we discuss new and notable candidates (e.g., *MYCN*, *EWSR1* retrogene, *MIR100HG*, and *MTMR9*), new evidence implicating one of multiple candidates at a locus (*TANGO2*), members of gene families that contain other genes known to be cancer drivers and prominent osteosarcoma pathways that are represented by multiple candidates.

### Nomination of 13 genes likely to be associated with risk

Aside from the fine mapping and limited functional support of the *CDKN2* locus [18], the other loci that are likely to implicate a specific gene/mechanism are those that have a single candidate within or near a mapped interval. The genes that are single candidates by any genomics criteria at a locus follow, with those that contribute to risk models underlined: *AQP4*, *OTX2*, *EWSR1* retrogene, *BMPER*, *MTMR7*, *MARCO*, *NELL1*, *FGF9*, *FBRSL1* and *BRINP3* (Fig. 2). We propose that those genes and *CDKN2A* (and possibly *CDKN2B*) and *IGF1* are likely to be mechanistically involved. The overlapping *CDKN2A* and *CDKN2B* genes and *ANRIL* are frequently deleted together in canine osteosarcoma. The first two of those have well established tumor suppressor effects relevant to osteosarcoma. However, there is additional evidence specifically implicating *CDKN2A* in osteosarcoma: somatic mutation in dogs [7] and germ line mutagenesis in mice [26]. *IGF1* and other dog size genes are mentioned because large body size is a major risk factor in canine osteosarcoma [14]. We also believe *MTMR9* and *TANGO2* (tier 1 in risk models) are likely to be functionally associated. Of our 13 priority genes, seven were present in the original annotation of GWAS intervals, 4 were prioritized by pathway enrichment and a transcriptional enhancer near *CDKN2A/B/ANRIL* was experimentally implicated.

**Fig. 3:**
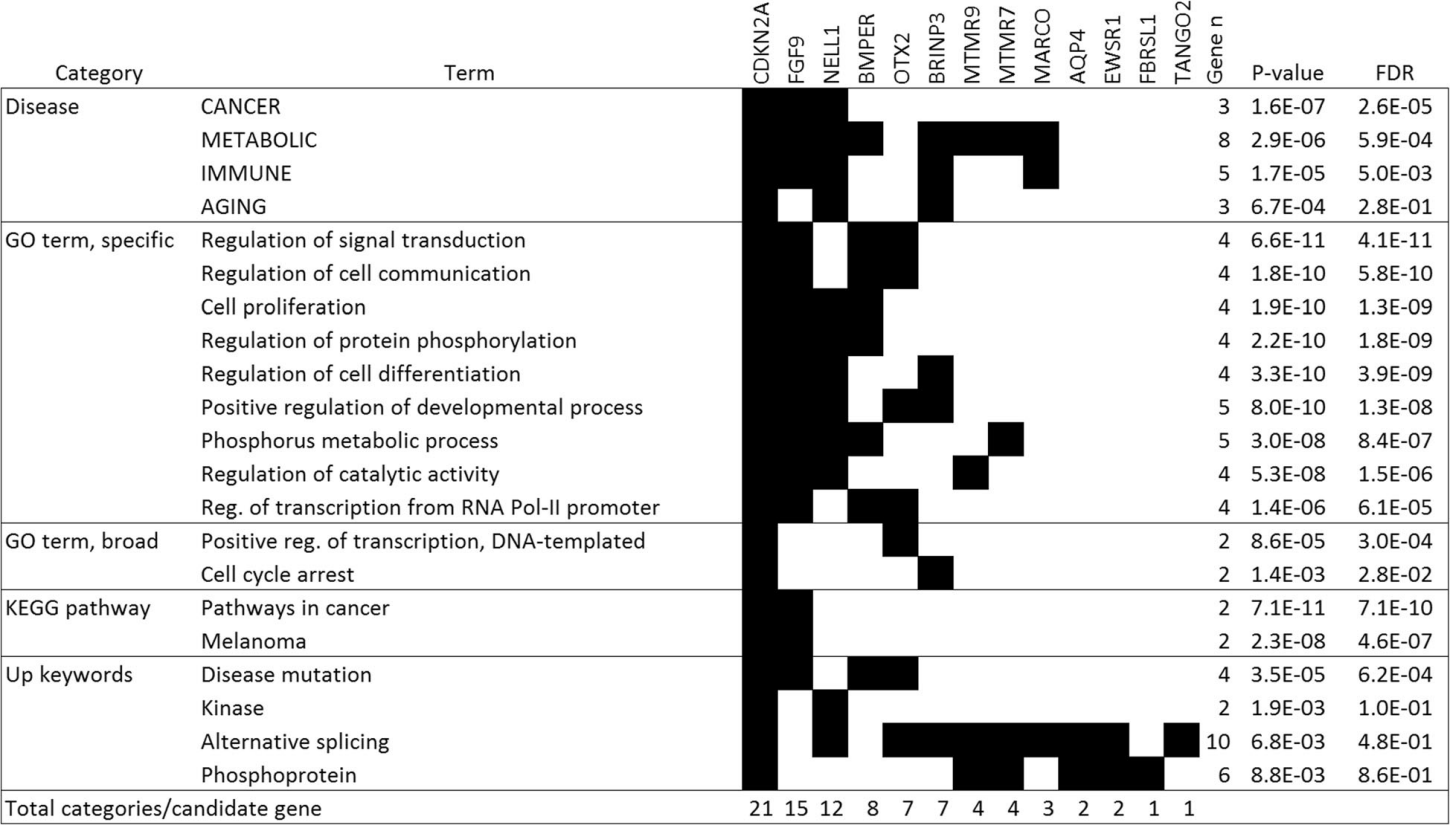
Pathway analysis of top canine osteosarcoma GWA candidates. Abridged pathway analysis of OS GWA candidates (*n* = 13), OS somatically-mutated genes (*n* = 23) and genes with germ line size-association (*n* = 7; implemented in DAVID). Select pathways show top significant terms for each category, except specific GO terms, which only shows non-redundant terms with FDR < 1E-4. See Supplementary Tables S4/5 for complete data

**Fig. 4:**
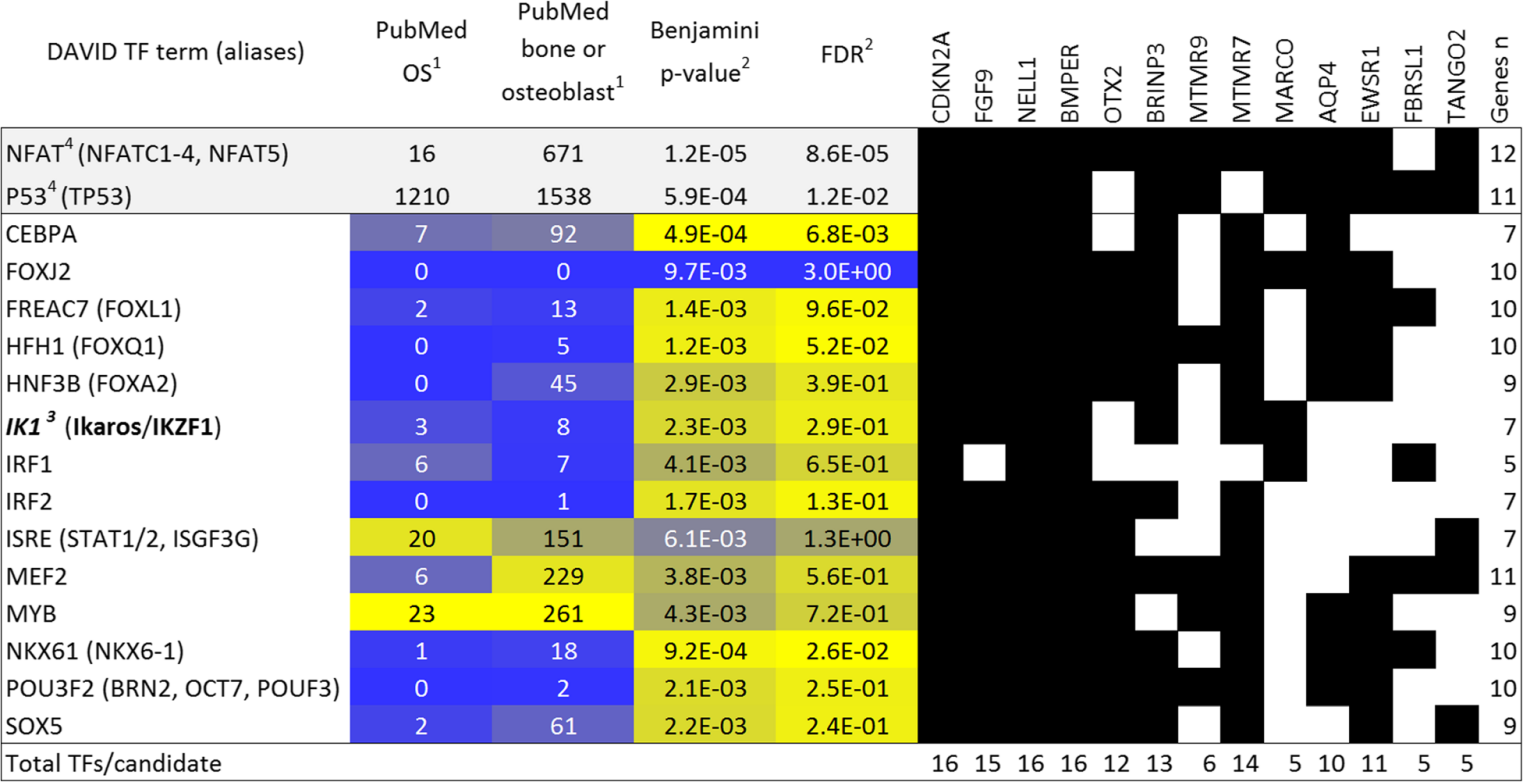
Transcription factor binding site analysis of osteosarcoma GWA candidate genes. Transcription factor binding sites shared by OS GWA candidates (*n* = 13), OS somatically-mutated genes (*n* = 23) and genes with germ line size-association (*n* = 7; implemented in DAVID). Legend: 1PubMed search with terms “osteosarcoma” or “bone or osteoblast, not marrow”; 2Multiplicity corrected *p*-value and False Detection Rate (FDR) in DAVID analysis; 3IKZF1 is a canine OS GWA candidate; color scale is yellow high, blue low, 4except the top 2 genes, which have dominant effects (NFAT and P53, gray)

Gene set analysis of the 13 priority candidates is very weakly powered, but achieved significance for two transcription factor binding sites (NFAT and FOXO1) and MTMR9 protein-protein interaction (Additional file 1: Text). To further consider the 13 genes, we combined them with the top 23 somatically altered genes in canine osteosarcoma and seven genes associated with dog size (Additional file 1: Text). The 43 genes showed enrichment for several relevant pathways (Fig. 3, Additional file 5: Table S4). Five of the 13 GWAS priority genes were associated with phosphorous metabolism, which is suggestive of bone biology. Six of the 13 represented one or more of the following pathways: cancer disease, disease mutation, cell proliferation, regulation of differentiation and cell cycle arrest. Lastly, the scavenger receptor *MARCO* is notable for suggesting a role in immunity. Analysis of the 43 genes for transcription factor binding sites (Fig. 4, Additional file 5: Table S5) showed many expected associations (incl. NFAT, P53 and MYB). In contrast, FOXJ2 only appears in a single osteosarcoma publication, but was positive for 10/13 priority genes (discussed in Additional file 1: Text). Seven of the 13 priority genes were positive for binding by Ikaros/IKZF1 binding. This is notable because *IKZF1* is the top ranked gene at the Irish Wolfhound chr18:1 Mb osteosarcoma risk locus (Fig. 2) and is one of the top contributors to risk in the modeling studies.

## Discussion

### Overview

The goal of this work was to ask if additional analyses of Karlsson et al.’s data [18] could accelerate clinical translation. Among our key results are discovery of the *FGF9* locus and validation of the original mapping in our own Greyhounds. Other major advances are prioritization of candidate loci according to effect sizes, and prioritization of candidate genes according to positional and biological relevance criteria. The results of risk-modeling prioritize 22 of the 34 mapped loci for breeding and veterinary translation. However, all loci can contribute important information for understanding osteosarcoma and developing therapies. Karlsson et al. reported 53 genes to be candidates within 31 loci and noted two additional loci with many genes each. 30 candidate genes were prioritized due to overrepresentation in enriched biochemical pathways. We updated and expanded the annotation of all 34 GWA-implicated loci to approximately 175 genes (75 correspond to the two loci simply annotated as many genes – unspecified – in the original study). We prioritized and ranked 151 of those based on published cross-species ‘omics datasets on osteosarcoma, bone development and known drivers in diverse cancer types. Below we propose that 13 specific genes are likely to be associated with osteosarcoma. Seven of those overlap Karlsson et al.’s 53 candidates and four overlap their 30 enriched-pathway genes.

### Risk modeling

The modeling studies showed forward selection method outperformed the LASSO and suggest considerations for future studies (Additional file 1: Text). Our models re-estimated the effect sizes of markers included in the models. These are not directly comparable to those of the original study [18]. Our ORs were estimated through a multiple logistic regression. They are therefore conditional to the fixed-levels for the other effects included in the model. In a genetically complex trait like osteosarcoma, locus contributions are unlikely to be independent of each other. Each individual’s risk is determined by a combination of many genetic and environmental factors – and their interactions. Presumably these effects cannot be individually isolated and summed to calculate total risk. We thus considered each marker’s effect together with those of all other markers. The following are some of the potential sources of uncertainty: interactions of small effects, removal of correlated markers (which could result from population structure) and bias in the population sampling. We caution that numeric values for effect sizes should not be given much weight for inference purposes. That is particularly important for markers that show quasi-complete separation, which we simply interpret as suggestive of large effects. Until proven otherwise, our OR values are only valid for the specific population sample that was used for their calculation. Even slight sampling changes such as modifying the case/control proportions can alter these values dramatically. Dog breed populations are not geographically or temporally uniform, and are subject to strong selective-mating. Here we mitigated such challenges of single-cohort studies by including a small independent cohort of Greyhounds and successfully validating that model. Before the present algorithms are implemented in breeding or for clinical use, further replication in independent cohorts and prospective testing of the models are indicated.

### Consideration of candidate genes and approaches to test them

Positional evidence of Mendelian traits has led to discovery of many hundreds of unanticipated molecular and biological mechanisms [17]. For human GWAS’s of complex traits, it is difficult to develop similar knowledge and utility because of the large number of loci and the minute effect of each. However, complex-trait variants with large effects make this feasible in dogs. That in turn facilitates diverse advances such as identification of gene-gene/−environment interactions and, ultimately, development of new therapies [9].

Here we expanded and updated Karlsson et al.’s published annotation. This information is not intended to be used as direct evidence of pathophysiological mechanisms. Rather, we believe the data can be used to identify functional variations and their effects. The annotation is primarily focused on protein coding genes because other types of genes and sequence elements can be challenging to annotate within and across species. We determined biological relevance of genes by their presence in many types of human, mouse and dog genome-wide datasets for osteosarcoma, bone development and genes strongly associated with cancer. There are many limitations to keep in mind with these analyses. This is similar to using literature searches to determine biological relevance of candidate genes. Major sources of such negative ascertainment bias include low or highly-variable gene expression levels. Another source is the type of genome wide data available, which favors that based on DNA and RNA, and almost completely omits proteomics, metabolomics, lipidomics, glycomics, etc. These various factors can affect the likelihood that any genic or regulatory sequence element is annotated. We provide the results of our systematic analysis as Fig. 2.

Almost all candidate genes reported here have an unknown status for contribution to osteosarcoma, but there is reason to prioritize many for consideration (incl. *AKT2, BCL2, EWSR1* retrogene and *MYCN* for their cancer notoriety). GWAS loci frequently deleted in canine osteosarcoma [18] implicate candidate genes such as the tumor suppressors *PPP1R13L* (encoding the TP53-inhibitor iASPP) and *PTPN18* (inhibitor of proliferation through the FGF/growth factor signaling/PI3K and RB/cell cycle pathways [27]). Other genes suggest roles in DNA damage, mutagenesis and repair (*ERCC1*/*2* and *NR2C1*). Haplotype phasing could facilitate fine mapping and identification of the functional variations (Additional file 1: Text). Without needing to know the functional variants, another approach is to screen all candidates in human cells or using in vitro or in vivo models in, say, frogs, fish or mice. For higher efficiency, this could be done on a highly-penetrant mutant background such as osteoblast lineage-specific knockout of TP53 or RB [26, 28]. This approach was successful in a mouse osteosarcoma model of *DLG2* loss [6]. Other candidates to consider alone or in combinations are in the linked PI3K and RB pathways (e.g., *MIR100HG*, *MYCN*, *AKT2*, *MTMR7*/*9*, *FGF9*, *PHLPP1* and *BRINP3*). Such models would reveal cancer mechanisms and the resulting patterns of somatic mutations. Therapies that were validated in model organisms could be followed by small genetically-targeted clinical trials in pet dogs. We predict this will dramatically improve the success rate of human trials and provide critical information about resistance mechanisms [4, 5].

### Therapeutic implications

Here we named 13 candidates likely to be the functional gene at their locus. *MTMR9* and *TANGO2* are located in loci which have one and two other candidates, respectively. The dual specificity phosphatase *MTMR9* would be implicated if the unlinked *MTMR7* were the functional gene at its locus. The two genes are expressed in osteoblasts and are known, in another cell type, to function coordinately to negatively regulate the insulin signaling/PI3K pathway [29]. Exome-sequencing in canine osteosarcoma showed *TANGO2* has the third highest rate of somatic sequence mutation [7]. Notably, this GWA locus was mapped in Rottweilers and the mutation frequencies were 3/22 in Golden Retrievers, 3/23 in Greyhounds, and 0/21 in Rottweiler. That suggests Rottweilers have the *TANGO2* pathway affected but through a germ line hypomorphic-allele. In humans, a germ line truncation variant of *TANGO2* is associated with increased risk of prostate cancer [30]. Previously unpublished survival analysis of TANGO2 protein expression in human prostate cancer is consistent with a tumor suppressor role (Additional file 1: Text). *MARCO*, one of our 13 priority genes with a moderate risk effect in our modeling studies, encodes a scavenger receptor that is an established checkpoint immunotherapy target [31]. That and the recent finding of frequent somatic mutation of the MHC class I receptor component B2M in canine cancer [7] have strong biomarker and therapeutic implications [31, 32].

Of our 13 priority genes, *FGF9* was the only one mapped by GWA meta-analysis of the three breeds together. The others were mapped in one breed; three of those are fixed in a second breed. *FGF9* has strong relevance to skeletal development and osteosarcoma. It participates in the coordination of osteo- and chondro-genesis, and is a potent negative regulator of longitudinal bone growth [33]. Specifically, FGF9 is secreted by osteoblasts and bound on their surface by FGFR1 and FGFR2, and on chondrocytes by FGFR3. It is a member of the most highly-implicated pathway shared by human and canine osteosarcoma, growth factor signaling/PI3K [7, 34]. *FGF9* was recently shown to have borderline genome wide significance in a melanoma GWAS [35]. The contribution of *FGF9* to osteosarcoma risk could also stem from its role in angiogenesis associated with long bone growth and repair [36]; or could be due to altered expression in the hypothalamus [37], which, in concert with the pituitary, is a master regulator of long bone growth [38]. It could have cell-autonomous effects on angiogenesis, migration and metastasis, or could play a chemotactic role through its expression in lungs [39].

Whereas all four FGF receptors are in the stringent Cancer Gene Census, no member of the ligand family has that status. A recent study of somatic sequence and structural mutations in canine osteosarcoma showed that focal amplifications spanning *FGFR2* are the most common of any oncogene [7]. 44% of 64 osteosarcoma tumors had focal amplification and 30 had deletion of *FGFR2*. Similarly in humans, FGF receptor genes are frequently amplified in different cancer types: including *FGFR1* and *FGFR2* in sarcomas and osteosarcoma [40]. *FGFR1* amplification in human osteosarcoma is associated with worse response to chemotherapy [41].

Kinase genetic dependencies for viability were recently screened by siRNA in 117 diverse types of human cancer cell lines (*n* = 714 kinases) [42]. Osteosarcomas were significantly enriched for a cluster of six skeletal-pathway kinases that includes FGFR1 and FGFR2. And osteosarcomas were significantly more sensitive to FGFR inhibitors than other cancer cell lines. Those effects were independent of receptor amplification status and persisted when *FGFR1*/*2*-amplified cell lines were excluded. The osteosarcoma selectivity of one compound was confirmed using published data on 660 tumor cell lines. In mice, a transposon-based forward genetic screen was performed to identify proto-oncogenes and tumor suppressors [26]. 232 common insertion sites were found, including in canine GWA candidates *Cdkn2a* and *Akt2*. *Fgfr2* was not a common insertion site, but was present in two of three mice (of 19 total with metastasis) that distinguish a group with intermediate levels of correlation between insertions in primary tumors and metastases (Figs. 8 and S8 in Ref. [26]). This is consistent with an angiogenesis role in metastasis, which would not rule out other roles such as in growth signaling. Thus, despite not contributing to our risk models, *FGF9* suggests an important mechanistic clue. It is also an attractive pharmacological target: it is a secreted protein and its receptors are expressed on the cell surface of osteoblasts/osteosarcoma cells. Given the putative germ line association of *FGF9* with melanoma risk in humans [18], our finding in dog osteosarcoma presents a valuable model in which to dissect and target the molecular mechanisms.

Karlsson et al. fine-mapped the risk interval near *CDKN2A/B/ANRIL* to 15 kb and nominated a functional variant that results in loss of binding by the bone transcription factor PAX5 [18]. The frequent deletion of this locus in dog osteosarcoma (72% of tumors [7]) hints the risk allele has an increased rate of loss [9]. Somatic mutation of *CDKN2A* in canine osteosarcoma also includes frequent sequence changes [7]. Whereas most tumor suppressor pathways seem difficult to target, *CDKN2A* suggests straightforward possibilities. INK4a/p16 (encoded by *CDKN2A*) is a pan cancer tumor suppressor with a major anti-cancer role of G1 cell cycle arrest by inhibition of CDK4/6 (as part of the Cyclin D arm of the cell cycle/RB/E2F pathway). There are several ongoing clinical trials of CDK4/6 inhibitors in human hormone-positive breast cancer and intense investigation of combination therapies [43]. Similar to other targeted cancer therapies, a prominent issue with CDK4/6 inhibition is drug resistance. For example, CDK4/6-inhibitor resistance in human gastric cancer was shown to be associated with the second arm of the cell cycle/RB/E2F pathway – Cyclin E and CDK2 [44]. As increased dog size is strongly associated with osteosarcoma risk [14], *IGF1* variation associated with size [45] and fixed in all three breeds should also be considered. Thus *CDKN2A/B*, *IGF1* and the two single candidates at their loci – *MTMR7*, *FGF9* – seem likely to be associated with canine osteosarcoma risk. *CDKN2A/B* and *MTMR7* contribute to osteosarcoma risk in our modeling studies, and *IGF1* does so by proxy with size.

## Conclusions

Our study shows how reanalyzing published data can yield new understanding. By conducting meta-analysis of Karlsson et al.’s osteosarcoma GWA data, we identified a new a candidate gene, *FGF9*. We then included all 34 peak markers to create breed-specific and general models of osteosarcoma risk. We showed 22 markers contributed to the risk models and validated the Greyhound model in a new cohort. Prospective studies are necessary to further validate the modeling, and to measure effect sizes in multiple samples. Assuming the models are correct, these 22 markers should be prioritized for breeding and clinical purposes. We updated and expanded the GWA gene annotation, and nominate 13 candidates to be likely culprits at their mapped loci. We argue there is sufficient evidence to conduct targeted clinical trials, including with CDK4/6 and FGFR1/2 inhibitors. In summary, these findings indicate how mouse models and canine translational investigations could rapidly accelerate and lead to new therapeutic approaches for dogs and humans.

## Methods

### Datasets and greyhound prediction model validation cohort

To perform our study, we use the association list and SNP data published by Karlsson and collaborators in 2013 [18]. For that study, a cohort of three dog breeds (Greyhound, Irish Wolfhound and Rottweiler) were genotyped using the Illumina 172 K Canine HD Array. These cohorts were a collection of cases and controls for dog osteosarcoma, Greyhounds where represented by 153 cases and 114 controls; Irish Wolfhounds by 28 cases and 62 controls; and Rottweilers by 80 cases and 55 controls. For more details on the subjects please refer to the original publication; the authors of the aforementioned publication provided their SNP genotype data and cases/control status designations.

A cohort of independent case/control Greyhounds was used only to validate the Greyhound models. This cohort consists of 12 cases and 24 controls collected by our research group. Samples were collected with the appropriate consent and animal care protocols (IACUC, protocol number 2010A0025-AM1). DNA was extracted from whole blood using a commercial DNA isolation kit (Puregene Genomic DNA purification kit, Gentra; with an additional ethanol precipitation step for optimal DNA quality). Extracted DNA samples were genotyped with the Illumina 172 K CanineHD Array following standardized methods.

### Intersection union testing meta-analysis across breeds

We performed a genome wide association test on the SNP genotype data of Karlsson et al. in combination with an Intersection Union Testing (IUT) step segregating IUT subgroups according to breed [19–22]. We included the IUT step to detect loci that were significantly detected on multiple breeds. We set significance thresholds at the Bonferroni corrected alpha calculated on the total number of valid IUT tests. We used Fisher’s combined *p*-values to evaluate each SNP position across breeds. The experimental design of this analysis is comparable to a meta-analysis across breeds where each breed is evaluated independently. Markers with a *p*-value equal or lower than the significance threshold was declared significant. Greyhounds, Rottweilers and Irish Wolfhounds were IUT-tested in all pairwise combinations and all three together. All genome wide association analyses were performed with PLINK v1.09 [46] using the association case/control test. The IUT and Manhattan plot visualizations were performed on SAS/STAT® v14.1 (SAS Institute, Cary NC).

### Remapping of candidate genes using new canine assembly data

Candidate loci reported in the original manuscript [18] were remapped using more recent annotation resources like the Broad Improved Canine Assembly v1 [23] in conjunction with additional data: regions with *S*_*i*_*/Di* statistics indicative of selection [47], and multi-breed genotype data [48] to identify the minimal overlap regions of the risk haplotype spanning the peak SNP at each locus. Assuming the risk variant precedes breed-creation, that minimal overlap region could be predicted to contain the functional variant. We generated that haplotype data as described previously, by direct phasing using Hayward et al.’s extensive breed-specific genotype data [49, 50]. *S*_*i*_*/Di* and haplotype phasing were visualized on the UCSC Genome Browser (canFam3.1; see Additional file 6: Data and Table S##).

### Predictive modeling

We used two different model selection methods to create parsimonious prediction models for the generalized sample and for each breed separately. For both, we fitted multiple logistic regressions of a Bernoulli (case/control status) dependent variable using the peak SNPs reported by Karlsson et al. and those detected in our IUT genome wide association meta-analysis. For both model-selection methods we extracted the data only for the markers to be analyzed and recoded them 0 for AA allele, 1 for AB allele and 2 for BB allele. We designated A and B as stated in the SNP *rs* accession number entry and used them in all models as class variables. The first model selection method we used was a Stepwise Forward selection without intercept and based on a 0.05 alpha inclusion and exclusion threshold. The exact logistic regression was approximated with a penalized likelihood method (Firth’s method) [51]. Briefly, the selection process began with a model with no terms. Independent variables were sequentially added based on their lowest *p*-values in the generalized model. Before adding the next term, the selection method would remove any variables that became non-significant after the inclusion of the previous term. The selection process was terminated when no more terms could be added or removed from the model. For the second selection method we used the LASSO (least absolute shrinkage and selection operator) method [52] on a model with no intercept and selection of the best model based on the best (lowest) Akaike’s Information Criterion (AIC). Briefly, the LASSO is a regularization method that shrinks certain coefficients using a penalty parameter (λ) that is optimized systematically until a specific criterion is achieved. All modeling was performed on SAS/STAT® v14.1. The first model selection method was performed using PROC LOGISTIC using the MISSING option on the CLASS statement to handle missing calls, and the NOINT and FIRTH options in the MODEL statement indicating no intercept fitting and implementation of Firth’s Method. The second model selection method was performed using PROC HPGENSELECT using the DIST = BINOMIAL and NOINT options in the MODEL statement to indicate logistic regression and no intercept fitting. The METHOD = LASSO (CHOOSE = AIC) option in the SELECTION statement was used to indicate the LASSO model and selection of the best model according to the lowest AIC. The LASSO selected model was then rerun to obtain all parameter estimates. This was done with the same procedure as the first model selection method (using PROC LOGISTIC), but for this rerun we removed the stepwise selection step.

We also explored the idea of interactive effects among our list of candidates. To do that we performed the epistatic interaction analysis implemented in PLINK v1.09. The interaction effect was tested for each pair-wise combination. Since the number of pairwise comparisons is considerable, we used a Bonferroni adjusted threshold to detect epistatic interactions. This was performed in each cohort and in the general cohort separately. None of the interaction effects passed the threshold and, thus, none were included in any model.

### Model validation with an independent greyhound cohort

Validation of the Greyhound models was done by fitting the same multiple logistic regression models selected in the model selection stage. We evaluated the performance of the models by fitting the same procedure (PROC LOGISTIC) in SAS/STAT v.14.1 using only the predictors selected by the model. We estimated the Max-Rescaled R^2^, performed a goodness of fit test, and evaluated the percent of correct calls. The latter values were used to determine how well the model performed.

### Inference in other breeds

We used genotype data from Hayward et al. [48] to evaluate the potential application of the modeling data to other breeds (besides the 3 evaluated in this study). We conducted hierarchical clustering analysis using the arithmetic average linkage method. We performed this analysis using allele frequencies over all pedigree breeds included in the data set, but only for those with at least 5 individuals. We excluded mixed breed and village dogs. It is important to note that this data set does not have osteosarcoma case/control status. Therefore, we base our inference on breed similarities based only on the set of markers we included. This analysis was performed on SAS/STAT 14.1 using PROC CLUSTER with the METHOD = AVERAGE option. PROC TREE was used to generate the dendrogram. Squared multiple correlations (R^2^) and average distance between clusters values were used to determine the cluster distances in the dendrogram.

## Additional files

Additional file 1: **Text.** Biological and translational relevance. (DOCX 224 kb)

Additional file 2: **Table S1.** Intersection Union Testing GWAS *p*-values (individual set, MaxP and Fisher’s Combined). (XLSX 42086 kb)

Additional file 3: **Table S2.** Risk modeling odds ratios calculated by Firth’s logistic regression. (XLSX 28 kb)

Additional file 4: **Figure S1.** Cluster analysis of frequencies of canine osteosarcoma GWAS risk alleles across breeds. **Figure S2.** EWSR1 and canine EWSR1 retrogenes multiple sequence alignment across diverse representative species. **Figure S3.** Alignment of ZF-RanBP domain and C-terminal Nuclear Localization Signal of EWSR1 and canine EWSR1 retrogenes with those of diverse other species, and with those of the other human proteins with the ZF-RanBP domain. (PDF 940 kb)

Additional file 5: **Table S3.** Updated and expanded annotation of canine osteosarcoma GWAS loci. **Table S4**. Gene list for biochemical pathway and transcription factor (TF) binding site analyses. **Table S5.** Full results of combined biochemical pathway and TF analyses of Table S4 genes (DAVID, Huang et al., PMID: 19033363; PMID: 19033363). **Table S5-trim.** Table S5 trimmed to only include categories associated with the 13 canine genes likely to be associated with germ line risk of osteosarcoma. (XLSX 214 kb)

Additional file 6: **Data.** Haplotype phasing across osteosarcoma GWAS risk alleles. (ZIP 162 kb)

## Abbreviations

BioGRID: Biological General Repository for Interaction Datasets
BMP: Protein family
BMP: Binding endothelial regulator
BMPER: Bone morphogenetic protein
chr: Chromosome
DAVID: Database for Annotation, Visualization and Integrated Discovery
EMMAX: Efficient Mixed-Model Association eXpedited
GWAS: Genome wide association studies
IUT: Intersection Union Test
LASSO: Least absolute shrinkage and selection operator
MTMR protein family: myotubularin related
OR: Odds ratio
SNP: Single nucleotide polymorphism genetic marker
TANGO protein family: Transport and golgi organization
