# Supplementary material for: Risk-modeling of dog osteosarcoma genome scans shows individuals with Mendelian-level polygenic risk are common
Source: BMC Genomics. 2019 Mar 19;20:226. doi: 10.1186/s12864-019-5531-6 (PMC6425649; doi:10.1186/s12864-019-5531-6)
Supplement: Supplementary file 1 — Text. Biological and translational relevance. (DOCX 224 kb) [file 12864_2019_5531_MOESM1_ESM.docx]

**Supplementary Text**

**Discussion**

**Osteosarcoma risk in dogs**

Large body size is a major risk factor for canine osteosarcoma (based on weight measures as proxy for size) [1, 2]. [This is true in humans [3], but difficult to measure due to socioeconomic issues [4]. Increased height has been and continues to be under evolutionary selection, and is affected by assortative mating [5]. Height is associated with wealth, and, in turn, social class and education [6]; all of those are mitigating factors in the risk, development and outcomes for cancer and diverse other diseases [4].] Calculation of dog osteosarcoma-risk odds ratios (ORs) using logistic regression and adjusting for body size (and other variables that had small effects), showed <23kg dogs had an OR of 1 compared to 2.5 for 23-33kg, 2.7 for 34-44kg and 2.8 for >45kg [2]. However, the raw ORs, which revealed the predominant size effect, were 1, 5.9, 10.3 and 22.8, respectively.

Epidemiological data indicate incidence rates are 13-27 fold higher in dogs vs. humans [1, 7]. As those estimates apply to the full dog population, we further define the osteosarcoma high-risk population here. For that we used American Kennel Club data for new registrations in 2008, focusing on a breed-standard weight list of 84 breeds [8] that account for ~90% of all registrations and include the most popular breeds. Almost exactly half of ~640,000 dogs were above or below 23kg. The mean breed-standard weight of the full population was 22kg; for the >23kg group it was 34kg (between osteosarcoma risk of OR=5.9 and 10.3); for the <23kg group it was 8kg (OR=1). Similar to the total pedigree dog population, shelter dogs have a mean weight of 18.4kb (based on 2009 US study of >2,700 dogs [9]).

Notably, there are great breed and even sub-breed effects in addition to standard size [7, 10-13]. For instance, the racing Greyhound (mean weight of 29.5kg) has an osteosarcoma prevalence of 6% [14], but the AKC “show” Greyhound – derived from the same population ~100 years ago – does not have similarly-elevated risk. The Whippet is very closely related to the Greyhound [13, 15] and was also selected for racing performance. However, the Whippet has a mean breed weight of 15.9kg and no elevated risk of osteosarcoma (see Whippet in cluster analysis in the main article text). Clearly, the prevalence of osteosarcoma in dogs is extremely variable and it is important to account for body size and breed. Based on the breed weight standards and population sizes, the incidence for the >23kg is twice that of the full dog population. We speculate that the group of breeds at the highest risk of osteosarcoma is likely to have on the order of a 10-fold higher incidence than dogs in general.

**Selection analyses and breed-specific phasing of risk-haplotypes**

We considered whether the 34 loci associated with canine osteosarcoma (Karlsson et al. [16] and this study) show evidence of selection under domestication. This was done using Vaysse et al.’s selection findings based on 509 dogs from 46 breeds, which included the three GWA breeds in this work: the *S_i_* statistic detects reduced heterozygosity and *D_i_* detects population differentiation (Suppl. Table S3) [17]. None of the breeds showed evidence of selection of the risk allele. This may seem surprising given that eight mapped loci were shown to be fixed for the risk allele in at least one other breed. However, *D_i_* would not be detected for haplotypes common in other breeds, and *S_i_* was calculated in 150kb windows and >250kb segments were strongly overrepresented in real vs. simulated detection (consistent with recent selection events) [18]. It is thus possible that finer-resolution analyses may reveal evidence of a selection sweep in domestication prior to modern breed creation. It seems unlikely that any of the GWA risk haplotypes in the present study have been under recent selection. However, that possibility cannot be ruled out. The issue is very important because it has long been speculated that selection under domestication explains the high prevalence of cancer in dogs (e.g., racing performance in Greyhounds). The other possibility is that some variants were selected in domestication, but prior to breed creation. This is consistent with the chr11:41Mb risk allele, which was not identified in wolves but has approximately 50% allele frequency across dog breeds [16].

Among the challenges in considering selection, each breed has approximately one quarter of its genome fixed for >100kb blocks, and each such haplotype could be the result of selection or genetic drift [16]. To further enhance the annotation, we determined breed-specific phased haplotypes using a genotype dataset of ~4,200 dogs from 150 breeds [19] (provided as a track for the UCSC genome browser, Suppl. Data). The methodology is described in the main article’s Methods section and illustrated in the following references [20, 21]. In the present case, this information is most useful in cases where multiple breeds with high osteosarcoma prevalence [22] share a risk-haplotype; the functional variant would be predicted to lie within the minimal interval of overlap across those breeds. This can also be tested by using our phasing data for selection of breeds that can be used in prospective studies of osteosarcoma risk. Such fine mapping could be achieved with as few as the two popular breeds with closest breakpoints in the ancestral-haplotype on either side of the peak marker at each GWA locus.

**Risk modeling**

Our logistic regression studies use two model selection methods that have been used for predictive modeling in diverse applications in oncology. The stepwise forward selection method has been used in cancer applications such as estimation of cancer prognosis [23], modeling of complex treatment patterns in a clinical cancer registry [24] and predictive models of cancer risk [25]. The cancer applications of the LASSO method are disease progression [26], identification of gene-gene interactions [27] and prediction of patient survival from transcriptomics data [28].

We believe the stepwise forward selection method outperformed the LASSO method in this study because they based their selection thresholds on two different parameter estimates, “p-values” for the Stepwise Forward selection and “AIC values” for the LASSO. These choices drive the algorithm in different directions because each of those values is linked to specific properties of the dataset analyzed. It is not straightforward to predict which will be more appropriate. Both low p-values and low AIC values can be hard to achieve when correlation among predictors and response is low. For this study, our intention was not to test the efficiency of the two methods. Rather, we asked if they could yield parsimonious models that had a satisfactory potential for clinical application. Method selection for model creation is debatable since outcomes cannot be simply classified as correct or incorrect without further studies. In addition, non-statistical conditions often limit practical applications. For example, here we prioritized clinical use and favored models with fewer parameters.

**Risk inference in other breeds**

Because osteosarcoma risk is highly variable across dog breeds, we conducted breed cluster analysis of only the risk alleles. Using a genotype dataset with ~4,200 dogs from over 150 breeds [29], we constructed a hierarchical cluster tree. Clustering was based on the correlations of the allele frequencies for the 34 risk loci (Suppl. Fig. S1). Our intention was to evaluate which breeds would be most related to the three index breeds at these risk loci. Rottweilers clustered with seven other breeds with at least 75% correlation among them: American Pit-bull Terrier, Staffordshire Bull Terrier, Cane Corso, Neapolitan Mastiff, Boston Terrier, Bouvier des Flandres and French Bulldog. That cluster mirrors their breed relationships [15, 18], but does not include the breeds most closely related to the Rottweiler in this analysis, such as the Giant Schnauzer, Doberman Pinscher and Great Dane [13, 15]. Greyhounds clustered along with Belgian Malinois and Newfoundlands, which lie outside the most closely related ~40 breeds, with a correlation of at least 65%. In contrast, the Whippet is genetically very-closely related to the Greyhound [13, 15], but here clusters outside of a supercluster that includes the majority of the members of most breed groups, except the ancient/Asian group. The Irish Wolfhound, which is also closely related to the Greyhound, did not cluster with other breeds and also lies outside that supercluster.

Therefore, the clustering by osteosarcoma risk allele frequencies differs from breed phylogenies based on the whole genome [15, 18]. This data can be used to test whether clustered breeds have similar osteosarcoma risk according to data from cancer registries [16, 22] or prospective studies. In the absence of such phenotype-genotype correlations, additional variation would be suspected. Karlsson et al. conducted a low powered 9-breed analysis of the Greyhound risk allele at chr11: 41Mb [16]. This locus contributed to osteosarcoma association in Leonbergers and Great Pyrenees, but not Labrador and Golden Retrievers, Great Danes or Mastiffs. That and our clustering results are consistent with our observation that the osteosarcoma risk haplotypes do not have signatures of recent selection.

**Biological relevance of osteosarcoma GWAS loci**

**Canine body size genetics**

As noted above, body size is strongly associated with osteosarcoma risk and has to be accounted for in the estimation of inherited risk. *IGF1* variation explains much of the reduced-size variance across all but giant dog breeds [30, 31] and is fixed for the non-small size allele in most large and giant breeds (Rottweilers appear to carry the small-size marker but not the phenotype). Several other gene variants associated with dog size [31] are also in that osteosarcoma-relevant biochemical pathway of growth factor signaling/PI3K (*IGF1R*, *GHR*, *IRS4* and *IGSF1*) or otherwise cancer relevant (*HMGA2*).

**Updated annotation**

Our annotation adds many new candidates and potential biological mechanisms (Fig. 2 and Table S3). Those include new identification of major cancer driver genes. *MYCN* lies 200kb outside an interval without a candidate gene in the original study. One locus has a single positional candidate that is a retrotransposed Ewing sarcoma gene *EWSR1* which is annotated to be actively expressed and frequently somatically-mutated in canine lymphoma (*EWSR1CR2*; see separate section below; Figs. S2/S3) [32]. Among our top candidates, *MTMR7* and *MTMR9* are non-linked genes of the myotubularin gene family. Myotubularins are dual-specificity phosphatases that act on phosphatidylinositol 3-phosphate and inositol 1,3-bisphosphate to mediate negative regulation of the insulin signaling/PIK3 pathway. The phosphatase domain is absent in MTMR9*,* but it functions by activating MTMR7 through hetero-association [33]. An interval mapped in one breed and fixed for the risk allele in another has two candidate protein coding genes, and *MIR100HG,* which is associated with several cancers in humans [34]*.* *MIR100HG* is a polycistronic spliced lincRNA gene that encodes the protein coding sequence *BLID* and the miRNAs *MIR100*, *MIR125B1*, and *LET7A2* in its third intron. *MIR100* and *MIR125B1* can have oncogenic and suppressive effects in different cancers, but increased levels of in osteosarcoma open biopsy samples were shown to be predictive of poor response to neoadjuvant chemotherapy ([35] and references within). *MIR100* is more abundantly expressed in highly proliferative osteosarcoma cell lines and experimental over-expression of both *MIR100* and *MIR125B1* in three osteosarcoma cell lines increased proliferation, invasiveness and resistance to multiple chemotherapies [35, 36]. In contrast, there is no clear evidence for *LET7A2* and the let-7 family is generally tumor suppressive.

*C22orf25*, now known as *TANGO2*, was reported in the original study. The same group subsequently found that *TANGO2* has the third highest rate of somatic sequence mutation in canine osteosarcoma (convolution testing p-value=1.86 x 10^-12^) [37]. Presumably due to the name change, this link between the two studies was not reported. TANGO proteins have roles in loading secretory-protein cargo in the endoplasmic reticulum [38], but studies of *TANGO2* suggest key roles in mitochondrial function [39]. Human germ line mutations in *TANGO2* result in infancy-onset recurrent metabolic crises with encephalocardiomyopathy [39, 40]. *SORL1*, the top of three candidates at chr5:12Mb, encodes another membrane trafficking protein that regulates protein exit from the endoplasmic reticulum [41]. This is reminiscent of a study of pediatric solid tumors that included transmission electron microscopy analysis [42]. The distinguishing subcellular features of osteosarcoma were swollen endoplasmic reticulum and collagen deposits. Such biology could relate to both endoplasmic reticulum stress and the effects of the extracellular matrix on metastasis.

Recent whole exome studies of families at high-risk of prostate cancer identified a *TANGO2* germ line variant segregating in one family and associated with increased risk in the general population (OR=1.65 95% CI=1.19 – 2.28; p=0.0025 [43]). While no mechanistic effects have been suggested for any cancer, we found that the Pathology Atlas at The Human Protein Atlas [44] has 176 TCGA prostate cancer samples that can be used for survival analysis with high quality immunohistochemistry of *TANGO2* using validated antibodies. Kaplan–Meier survival analysis show lower expression of *TANGO2* is associated with decreased survival (p=3.0x10^-4^). The five-year survival rates for the higher and lower expression groups were 43% and 14%, respectively. *TANGO2* is thus a candidate tumor suppressor gene. The limited but strong evidence of biochemical and subcellular localization properties hints at possible roles in apoptosis [38-40]. These could be in mitochondria or at their interphase with endoplasmic reticulum stress [45].

As we mentioned previously [10], many canine osteosarcoma GWA candidate genes are not known to be cancer drivers, but have family members that are (Suppl. Table S3). We also noted that several cancer driver pathways that are implicated by multiple candidate genes: cell cycle/RB/E2F (*CDKN2A*, *MYCN, BRINP3* and *MIR100HG*); growth signaling /PI3K (*AKT2*, *MTMR7*/*9*, *FGF9*, *PHLPP1*, *MYCN* and presumably fixation of non-small allele of *IGF1*); genome instability (*NR2C1*, *USP44*, *ERCC1/2, EWSR1* retrogene); apoptosis (*BCL2*, *SVIL*, *PPP1R13L*); cell migration (*TNFRSF11A, PHLPP1*, *SVIL*); TP53 (*CDKN2B*, *PPP1R13L*) and cell stemness, senescence or osteoblast differentiation (*KIAA1468*/*RELCH*, *MIR100HG*, *NR2C1*).

**Biological relevance: gene set analysis**

Gene set analysis of the 13 priority candidates is very weakly powered. Using DAVID, the only categories that are significant after multiplicity correction are NFAT and FOXO1 transcription factor binding sites, and MTMR9 protein-protein interaction (MTMR7/9 and EWSR1 by yeast two-hybrid; data source, BioGRID). NFAT is a key mediator of calcineurin signaling in bone development, including in osteoblasts, and is central to FGF, RANKL (implicated by *FGF9* and *TNFRSF11A/RANK* GWA candidates here) and WNT signaling [46]. FOXO1 is very prominent in bone development, including as the final mediator of PI3K/AKT pathway signaling that serves as the Go-No Go point for bone growth [47]. It also has roles in autophagy, apoptosis and stemness, and is highly sensitive to hypoxia, oxidative stress, vitamin D and energy metabolism.

In order to conduct some measure of pathway analysis, we combined the 13 likely candidates with the top 23 somatically altered genes in canine osteosarcoma and seven genes with germ line association with dog size (Suppl. Tables S4/5). In this way, we mitigate the lack of power by combining the 13 candidates we are agnostic about (30% of the 43 genes) with genes strongly associated with canine osteosarcoma risk and development. The one link suggestive of bone relevance is that five of the 13 priority candidates are members of the phosphorus metabolic process (corrected p-value=3 x 10^-8^, FDR=8 x 10^-7^, analysis n=43 genes; Fig. 4). Given that tissue of origin is the strongest molecular signature across cancers [42], this supports our nomination of these 13 priority genes. Disease categories are also promising, including – in ranked order – cancer (3 of 13 candidates), metabolic (8), immune (5) and aging (3). Among the top biochemical pathways are cell cycle arrest, proliferation, differentiation and positive regulation of development. The scavenger receptor gene *MARCO* shows some specificity for immunity in our pathway analysis. It is predominantly expressed in macrophages and dendritic cells, and is a proven target for immunotherapy [48].

The transcription factor binding site analysis showed NFAT as the top hit (p-value=5 x 10^-4^, FDR=8 x 10^-5^; analysis n=43 genes), followed by CEBPA and TP53 – which had binding sites in 12, 7 and 11 of the 13 likely candidates, respectively (Fig. 5); all three have major roles in osteoblast/osteosarcoma biology. Others highly implicated in osteosarcoma are MYB and ISRE (i.e., STAT1/2/ISGF3G complex), followed by IRF1 and MEF2B/C/D. Ikaros (encoded by *IKZF1,* called *IK1* in DAVID) is involved in osteoclastogenesis [49] – as is our GWAS candidate *RANK*/*TNFRSF11A* – and has less clear roles in osteoblasts [50]. Notably, *IKZF1* is the top of five candidates in the Irish Wolfhound chr18:1Mb locus, which is tier 2 in our risk modeling. This hints *IKZF1*, if associated with osteosarcoma risk, could have a role in immunity. FOXJ2 was positive for 10/13 priority genes, but the only appears in one published study of osteosarcoma (NCBI PMC database of full text articles). This work showed that FOXJ2 is a member of the cluster with by far the strongest effect size in analyses of miRNA and transcription factor networks. That subnetwork is associated with signaling and is described by its members miR-138, MYC, FOXC1, USF1, ZIC1 and FOXJ2. The authors propose this subnetwork is a key player in the RB1 pathway that regulates proliferation and cell cycle arrest.

**Ewing sarcoma EWSR1 retrogene**

The Greyhound Chr13:11Mb locus had no annotated genes in the original study. Here we find it to be a tier 2 locus in the modeling analysis, show it is fixed in Irish Wolfhounds, and identify a new gene within the mapped interval. That gene *ENSCAFG00000000749* is a retrotransposed Ewing sarcoma gene *EWSR1* with multiple introduced non-canonical introns (Suppl. Fig. S1). It is currently annotated as an active protein coding gene by both EnsEMBL (ENSCAFG accession ID above) and the Broad Institute Vertebrate Genome Biology group [51]. The latter group used their own experimental RNA (cDNA) sequencing data from eight dog tissues to define the dog transcriptome. Seven tissues showed poly-adenylation-enriched mRNA expression of this gene: brain, heart, kidney, liver, blood, ovary, skin and testis were positive; skeletal muscle was negative. Six of those seven tissues had RNA sequence that spanned an intron. Although the actual sequences are not accessible, we checked the genome alignments for two: TCONS 00012145 and 00009161. Both uniquely map to this gene and not to related sequences. Despite that supporting data, we have not found conclusive evidence describing the mRNA or protein, and we consider it an open question whether this is an active gene and, if so, what splicing patterns occur in vivo. However, the DNA/protein sequences have many changes from the ancestral *EWSR1* and both of these types of evidence have been reported. The protein is robustly expressed in the dog cell line MCDK according to mass spectroscopy (protein encoded by mRNA *ENSCAFT00000001155*; [52]). The dog gene is annotated as protein coding and having partial sequence evidence that it is expressed in several tissues (Broad improved genome annotation, UCSC Genome Browser, hub tracks; [51]). It was also reported to be expressed in fox pituitary [53]. BLAST analysis of the gene sequence and manual examination of that genome locus in three other carnivores with available genome assemblies – cat, ferret and panda – show the retroposition event is specific to the canine lineage.

Supplementary Figure S2 shows a multiple sequence alignment of the EWSR1 retrogene protein with that of the parent gene from three carnivores and divergent vertebrates. This analysis indicates the protein sequence is generally conserved over its full length, but is approximately as divergent from the mammalian consensus as the proteins from birds or amphibians. Its prototypical RNA binding domain RRM_EWS is a perfect match to the consensus over 84 amino acids, with the exception of one conservative substitution of valine to leucine. However, the protein sequence has three variants with strong functional implications. First, the “SYGQ2” sequence within the N-terminal transcriptional activation domain is absent. SYGQ2 mediates the GGAA-repeat binding and transcriptional regulation that is understood to mediate Ewing sarcoma [54]. Experimental fusion of the 64-amino acid SYGQ2 sequence to the Ewing sarcoma ETS partner FLI1 phenocopies full EWS-FLI1 fusions. Two other EWSR1 sequence elements are highly conserved with the exception of two key residues each (Suppl. Fig. S2). The zinc finger single-stranded RNA-binging domain zf-RanBP is conserved except for the third and fourth of the four cysteines that coordinate zinc the zinc finger. The Nuclear Localization Signal (NLS) motif – the C-terminal 10-amino acids – is conserved across EWSR1 of vertebrates its paralog FUS. In contrast, the canine *EWSR1* retrogene has two non-conservative substitutions at positions that are not only conserved in EWSR1/FUS, but also in the other paralog, TAF15. These findings suggest that the EWSR1 canine retrogene (*EWSR1CR2*) generally has the same general biochemical functions as EWSR1, but is not constrained by all of the latter’s ancestral roles. In other words, at least some molecular interactions mediated by the zf-RanBP and NLS sequences have changed in terms of partners or kinetics. Curiously, this gene was found to be one of the most frequently somatically-mutated genes in B-cell lymphoma in two dog breeds (it ranked #38 of 77 significant genes; [32]). All eight somatic mutations in protein coding sequence were missense changes; and five reverted to the amino acid sequence of canine EWSR1. The methods of that study suggest this cannot be due to incorrect mapping of short-read sequencing (normal vs. tumor per subject; 75bp paired-end sequencing; minimum of 10 overlapping reads required for a call). Another possibility is that the revertant mutations were the result of somatic gene conversion with the primary *EWSR1* gene (on another chromosome). Most of those somatic variants have flanking variation nearby that suggests gene conversion tracts would be short. This is consistent with experimental studies that show conversion tracts are overwhelmingly short – in one of those, 90% of 162 events had tracts <300bp [55]. [Note the ERRC1/2 proteins encoded in the Greyhound chr1:110Mb GWAS locus have roles in gene conversion [56].]

We are not aware of any *EWSR1* retrogene reported in another species. However, we previously identified copy number variation that spans another canine *EWSR1* retrogene, *ENSCAFG00000012292*, on a third chromosome [57]. We now refer to it as *EWSR1CR1* (Suppl. Fig. S2). Although it is classified as a pseudogene in EnsEMBL and in the updated dog genome annotation, the latter resource has RNA sequencing evidence that it may be expressed [51]. It was also reported to be expressed in the fox [53]. Among the suggestions it is a pseudogene, it contains a one-base insertion in the open reading frame. Our analysis of that site revealed a potential programmed ribosomal -1 frameshifting sequence and structure: a “slippery” heptamer of the type XXXYYYZ, where each letter is a different base, immediately followed by a stem looping sequence. Further analyses are necessary to determine the status of this gene. The *EWSR1CR1* locus has been shown to have signal of selection under domestication in Greyhounds and Elkhounds (due to reduced heterozygosity and population differentiation; [18]). Common genomic alterations spanning this locus have been reported in canine melanoma [58] and lung cancer [59]. Thus, if it were shown to be an active gene it should be considered for association with traits of domestication and cancer risk.

It is unclear why Ewing sarcoma is not known to occur in any other species. Ewing sarcoma is predominantly caused by fusion of the N-terminal, transcriptional activating, half of EWSR1 and ETS transcription factors (most commonly FLI1, then ERG, but also others). Arguably the leading hypothesis hinges on the GGAA-repeat sequences bound by the N-terminal region of EWSR1 and comprising the middle part of the ETS binding consensus. *EWSR1-ETS* fusions have cell type specific effects [60]. Sequences that are bound by the fusion and mediate gene repression show robust DNA conservation, but those that mediate activation are not. These facts can be interpreted to suggest that the uniqueness of these fusions causing Ewing sarcoma – but not other cancers – is due to cell type specificity (but see similar epigenetic effects in mesenchymal stem cells in ref. [61]); and the absence of GGAA-repeat conservation accounts for uniqueness of Ewing sarcoma existing in humans but not in any other studied species. Here we find support for the latter regarding canine *NR0B1*, which is required to be induced for human Ewing sarcoma. The nearest GGAA sequences repeated at least four times are ~650kb downstream (repeat n=18) and ~2Mb upstream (n=1) (data not shown). At least seven carefully designed attempts to create a genetic mouse model of Ewing sarcoma have failed [62]. However, the unique properties of EWSR1 fusions and the animal species in which they result in Ewing sarcoma may not be that simple. Introduction of an *EWSR1-ETS* fusion into mouse osteochondrogenic progenitor cells induces Ewing sarcoma-like cancer [63]. The authors of that work speculate that the rarity of the human condition is a reflection of the relative rarity of the requisite progenitor cells. This may be generally true, but could also be affected by species-specific genetics or environmental effects. For example, why is osteosarcoma rare in humans but very common in large dogs?

GWAS’s of human breast and pancreatic cancer have shown the *EWSR1* locus is associated with risk [64-66]. While that is only positional evidence at this time, a potent oncogenic role of non-fused *EWSR1* was recently shown in a subtype of prostate cancer. That form is associated with four ETS-family cancer driver genes and wild type (non-fused) *EWSR1* and [67]. In humans, only those four, of 21 total ETS family members, have protein-protein interactions with EWSR1. That interaction and the co-expression of *EWSR1* are required for various cancer phenotypes. That applies to the ETS-driven subtype of prostate cancer, but not the mutant KRAS-driven subtype. These new findings of wild type *EWSR1* cancer functions outside of Ewing sarcoma are interesting in light of our *EWSR1* retrogene findings. Although that locus was mapped in Greyhound and is fixed in Irish Wolfhounds, it may still be worth noting that the Rottweiler chr15:35Mb locus (which also contributes to our risk models) contains *ELK3*, a member of the ETS gene family.

If the *EWSR1* retrogene were associated with osteosarcoma, we would predict that otherwise-non-functional GGAA repeats become enhancers or repressors for osteosarcoma drivers. It also seems likely that normal activity of transcription factors critical to bone development – such as RUNX2 – could be affected. For instance, the transcription factor gene Osterix/*SP7* is directly bound and activated by RUNX2 to mediate osteoblast differentiation. Our analysis of mouse genome-wide RUNX2 binding in mesenchymal cells with and without osteogenic induction [68] shows two major peaks 5’ (~10kb away) and 3’ of the gene and a smaller intronic peak, and GGAA repeats of n=10 at ~8kb from 5’-end and intronically. The dog *SP7* gene has 16 GGAA repeats ~2kb upstream of the transcription start site. By contrast, the nearest GGAA repeats to human *SP7* lie almost 300kb upstream and 500kb downstream, and have many other genes between them. These observations support the notion that GGAA microsatellite sequences are incidentally associated with genes, but can have species-specific relevance to Ewing’s sarcoma.

**References**

1. Simpson S, Dunning MD, de Brot S, Grau-Roma L, Mongan NP, Rutland CS: **Comparative review of human and canine osteosarcoma: morphology, epidemiology, prognosis, treatment and genetics**. *Acta veterinaria Scandinavica* 2017, **59**(1):71.

2. Ru G, Terracini B, Glickman LT: **Host related risk factors for canine osteosarcoma**. *Vet J* 1998, **156**(1):31-39.

3. Mirabello L, Pfeiffer R, Murphy G, Daw NC, Patino-Garcia A, Troisi RJ, Hoover RN, Douglass C, Schuz J, Craft AW *et al*: **Height at diagnosis and birth-weight as risk factors for osteosarcoma**. *Cancer Causes Control* 2011, **22**(6):899-908.

4. Plomin R, Deary IJ: **Genetics and intelligence differences: five special findings**. *Mol Psychiatry* 2015, **20**(1):98-108.

5. Joshi PK, Esko T, Mattsson H, Eklund N, Gandin I, Nutile T, Jackson AU, Schurmann C, Smith AV, Zhang W *et al*: **Directional dominance on stature and cognition in diverse human populations**. *Nature* 2015, **523**(7561):459-462.

6. von Hinke Kessler Scholder S, Davey Smith G, Lawlor DA, Propper C, Windmeijer F: **Child height, health and human capital: Evidence using genetic markers**. *Eur Econ Rev* 2013, **57**:1-22.

7. Rowell JL, McCarthy DO, Alvarez CE: **Dog models of naturally occurring cancer**. *Trends Mol Med* 2011, **17**(7):380-388.

8. Plassais J, Rimbault M, Williams FJ, Davis BW, Schoenebeck JJ, Ostrander EA: **Analysis of large versus small dogs reveals three genes on the canine X chromosome associated with body weight, muscling and back fat thickness**. *PLoS Genet* 2017, **13**(3):e1006661.

9. DeLeeuw JL: **Animal shelter dogs: Factors predicting adoption versus euthanasia**. Wichita, Kansas, US: Doctoral Dissertation, Wichita State University; 2010.

10. Alvarez CE: **Naturally Occurring Cancers in Dogs: Insights for Translational Genetics and Medicine**. *ILAR Journal* 2014, **55**(1):16-45.

11. Dodman NH, Moon R, Zelin M: **Influence of owner personality type on expression and treatment outcome of dominance aggression in dogs**. *J Am Vet Med Assoc* 1996, **209**(6):1107-1109.

12. Ostrander EA, Wayne RK, Freedman AH, Davis BW: **Demographic history, selection and functional diversity of the canine genome**. *Nat Rev Genet* 2017, **18**(12):705-720.

13. Parker HG, Dreger DL, Rimbault M, Davis BW, Mullen AB, Carpintero-Ramirez G, Ostrander EA: **Genomic Analyses Reveal the Influence of Geographic Origin, Migration, and Hybridization on Modern Dog Breed Development**. *Cell reports* 2017, **19**(4):697-708.

14. Lord LK, Yaissle JE, Marin L, Couto CG: **Results of a web-based health survey of retired racing Greyhounds**. *Journal of veterinary internal medicine* 2007, **21**(6):1243-1250.

15. vonHoldt BM, Pollinger JP, Lohmueller KE, Han E, Parker HG, Quignon P, Degenhardt JD, Boyko AR, Earl DA, Auton A *et al*: **Genome-wide SNP and haplotype analyses reveal a rich history underlying dog domestication**. *Nature* 2010, **464**(7290):898-902.

16. Karlsson E, Sigurdsson S, Ivansson E, Thomas R, Elvers I, Wright J, Howald C, Tonomura N, Perloski M, Swofford R *et al*: **Genome-wide analyses implicate 33 loci in heritable dog osteosarcoma, including regulatory variants near CDKN2A/B**. *Genome Biology* 2013, **14**(12):R132.

17. Vaysse A, Ratnakumar A, Derrien T, Axelsson E, Rosengren Pielberg G, Sigurdsson S, Fall T, Seppala EH, Hansen MS, Lawley CT *et al*: **Identification of genomic regions associated with phenotypic variation between dog breeds using selection mapping**. *PLoS Genet* 2011, **7**.

18. Vaysse A, Ratnakumar A, Derrien T, Axelsson E, Rosengren Pielberg G, Sigurdsson S, Fall T, Seppälä EH, Hansen MST, Lawley CT *et al*: **Identification of Genomic Regions Associated with Phenotypic Variation between Dog Breeds using Selection Mapping**. *PLoS Genet* 2011, **7**(10):e1002316.

19. Hayward JJ, Castelhano MG, Oliveira KC, Corey E, Balkman C, Baxter TL, Casal ML, Center SA, Fang M, Garrison SJ *et al*: **Complex disease and phenotype mapping in the domestic dog**. *Nat Commun* 2016, **7**.

20. Zaldivar-Lopez S, Rowell JL, Fiala EM, Zapata I, Couto CG, Alvarez CE: **Comparative genomics of canine hemoglobin genes reveals primacy of beta subunit delta in adult carnivores**. *BMC Genomics* 2017, **18**(1):141.

21. Zapata I, Serpell JA, Alvarez CE: **Genetic mapping of canine fear and aggression**. manuscript submitted for publication.

22. Gruntzig K, Graf R, Boo G, Guscetti F, Hassig M, Axhausen KW, Fabrikant S, Welle M, Meier D, Folkers G *et al*: **Swiss Canine Cancer Registry 1955-2008: Occurrence of the Most Common Tumour Diagnoses and Influence of Age, Breed, Body Size, Sex and Neutering Status on Tumour Development**. *Journal of comparative pathology* 2016, **155**(2-3):156-170.

23. Millan-Rodriguez F, Chechile-Toniolo G, Salvador-Bayarri J, Palou J, Vicente-Rodriguez J: **Multivariate analysis of the prognostic factors of primary superficial bladder cancer**. *J Urol* 2000, **163**(1):73-78.

24. Schmidtmann I, Elsasser A, Weinmann A, Binder H: **Coupled variable selection for regression modeling of complex treatment patterns in a clinical cancer registry**. *Stat Med* 2014, **33**(30):5358-5370.

25. Kim Y, Kwon MS, Choi Y, Yi SG, Namkung J, Han S, Kwon W, Kim SW, Jang JY, Kim H *et al*: **Comparative studies for developing protein based cancer prediction model to maximise the ROC-AUC with various variable selection methods**. *Int J Data Min Bioinform* 2016, **16**(1):64-76.

26. de Maturana EL, Picornell A, Masson-Lecomte A, Kogevinas M, Marquez M, Carrato A, Tardon A, Lloreta J, Garcia-Closas M, Silverman D *et al*: **A Bayesian Lasso Genome-Wide Multimarker Approach to Predict Prognosis: An Application to Bladder Cancer Progression**. *Human Heredity* 2015, **80**(3):117-117.

27. Sehl ME, Duncan BN, Ganz PA, Hussain SK, Zhang ZF, Lange KL, Sinsheimer JS: **Lasso Penalized Regression as a Screening Tool to Identify DNA Repair SNP-SNP Interactions in Familial Breast Cancer**. *Genetic Epidemiology* 2009, **33**(8):769-769.

28. Datta S, Le-Rademacher J, Datta S: **Predicting patient survival from microarray data by accelerated failure time modeling using partial least squares and LASSO**. *Biometrics* 2007, **63**(1):259-271.

29. Hayward JJ, Castelhano MG, Oliveira KC, Corey E, Balkman C, Baxter TL, Casal ML, Center SA, Fang M, Garrison SJ *et al*: **Complex disease and phenotype mapping in the domestic dog**. *Nat Commun* 2016, **7**:10460.

30. Sutter NB, Bustamante CD, Chase K, Gray MM, Zhao K, Zhu L, Padhukasahasram B, Karlins E, Davis S, Jones PG *et al*: **A Single IGF1 Allele Is a Major Determinant of Small Size in Dogs**. *Science* 2007, **316**(5821):112-115.

31. Rimbault M, Beale HC, Schoenebeck JJ, Hoopes BC, Allen JJ, Kilroy-Glynn P, Wayne RK, Sutter NB, Ostrander EA: **Derived variants at six genes explain nearly half of size reduction in dog breeds**. *Genome Res* 2013, **23**(12):1985-1995.

32. Elvers I, Turner-Maier J, Swofford R, Koltookian M, Johnson J, Stewart C, Zhang CZ, Schumacher SE, Beroukhim R, Rosenberg M *et al*: **Exome sequencing of lymphomas from three dog breeds reveals somatic mutation patterns reflecting genetic background**. *Genome Res* 2015, **25**(11):1634-1645.

33. Mochizuki Y, Majerus PW: **Characterization of myotubularin-related protein 7 and its binding partner, myotubularin-related protein 9**. *Proc Natl Acad Sci U S A* 2003, **100**(17):9768-9773.

34. Lu Y, Zhao X, Liu Q, Li C, Graves-Deal R, Cao Z, Singh B, Franklin JL, Wang J, Hu H *et al*: **lncRNA MIR100HG-derived miR-100 and miR-125b mediate cetuximab resistance via Wnt/beta-catenin signaling**. *Nature medicine* 2017, **23**(11):1331-1341.

35. Kubota D, Kosaka N, Fujiwara T, Yoshida A, Arai Y, Qiao Z, Takeshita F, Ochiya T, Kawai A, Kondo T: **miR-125b and miR-100 Are Predictive Biomarkers of Response to Induction Chemotherapy in Osteosarcoma**. *Sarcoma* 2016, **2016**:1390571.

36. Poos K, Smida J, Nathrath M, Maugg D, Baumhoer D, Korsching E: **How microRNA and transcription factor co-regulatory networks affect osteosarcoma cell proliferation**. *PLoS Comput Biol* 2013, **9**(8):e1003210.

37. Sakthikumar S, Elvers I, Kim J, Arendt ML, Thomas R, Turner-Maier J, Swofford R, Johnson J, Schumacher SE, Alfoldi J *et al*: **SETD2 Is Recurrently Mutated in Whole-Exome Sequenced Canine Osteosarcoma**. *Cancer research* 2018, **78**(13):3421-3431.

38. Saito K, Chen M, Bard F, Chen S, Zhou H, Woodley D, Polischuk R, Schekman R, Malhotra V: **TANGO1 facilitates cargo loading at endoplasmic reticulum exit sites**. *Cell* 2009, **136**(5):891-902.

39. Kremer LS, Distelmaier F, Alhaddad B, Hempel M, Iuso A, Kupper C, Muhlhausen C, Kovacs-Nagy R, Satanovskij R, Graf E *et al*: **Bi-allelic Truncating Mutations in TANGO2 Cause Infancy-Onset Recurrent Metabolic Crises with Encephalocardiomyopathy**. *Am J Hum Genet* 2016, **98**(2):358-362.

40. Lalani SR, Liu P, Rosenfeld JA, Watkin LB, Chiang T, Leduc MS, Zhu W, Ding Y, Pan S, Vetrini F *et al*: **Recurrent Muscle Weakness with Rhabdomyolysis, Metabolic Crises, and Cardiac Arrhythmia Due to Bi-allelic TANGO2 Mutations**. *Am J Hum Genet* 2016, **98**(2):347-357.

41. Schmidt V, Sporbert A, Rohe M, Reimer T, Rehm A, Andersen OM, Willnow TE: **SorLA/LR11 regulates processing of amyloid precursor protein via interaction with adaptors GGA and PACS-1**. *The Journal of biological chemistry* 2007, **282**(45):32956-32964.

42. Stewart E, Federico SM, Chen X, Shelat AA, Bradley C, Gordon B, Karlstrom A, Twarog NR, Clay MR, Bahrami A *et al*: **Orthotopic patient-derived xenografts of paediatric solid tumours**. *Nature* 2017, **549**(7670):96-100.

43. Karyadi DM, Geybels MS, Karlins E, Decker B, McIntosh L, Hutchinson A, Kolb S, McDonnell SK, Hicks B, Middha S *et al*: **Whole exome sequencing in 75 high-risk families with validation and replication in independent case-control studies identifies TANGO2, OR5H14, and CHAD as new prostate cancer susceptibility genes**. *Oncotarget* 2017, **8**(1):1495-1507.

44. Colwill K, Renewable Protein Binder Working G, Graslund S: **A roadmap to generate renewable protein binders to the human proteome**. *Nature methods* 2011, **8**(7):551-558.

45. Theurey P, Rieusset J: **Mitochondria-Associated Membranes Response to Nutrient Availability and Role in Metabolic Diseases**. *Trends Endocrinol Metab* 2017, **28**(1):32-45.

46. Sitara D, Aliprantis AO: **Transcriptional regulation of bone and joint remodeling by NFAT**. *Immunological reviews* 2010, **233**(1):286-300.

47. Kousteni S: **FoxO1: a molecule for all seasons**. *Journal of bone and mineral research : the official journal of the American Society for Bone and Mineral Research* 2011, **26**(5):912-917.

48. Georgoudaki AM, Prokopec KE, Boura VF, Hellqvist E, Sohn S, Ostling J, Dahan R, Harris RA, Rantalainen M, Klevebring D *et al*: **Reprogramming Tumor-Associated Macrophages by Antibody Targeting Inhibits Cancer Progression and Metastasis**. *Cell reports* 2016, **15**(9):2000-2011.

49. Miyamoto T, Ohneda O, Arai F, Iwamoto K, Okada S, Takagi K, Anderson DM, Suda T: **Bifurcation of osteoclasts and dendritic cells from common progenitors**. *Blood* 2001, **98**(8):2544-2554.

50. Son E, Do H, Joo HM, Pyo S: **Induction of alkaline phosphatase activity by L-ascorbic acid in human osteoblastic cells: a potential role for CK2 and Ikaros**. *Nutrition* 2007, **23**(10):745-753.

51. Hoeppner MP, Lundquist A, Pirun M, Meadows JRS, Zamani N, Johnson J, Sundström G, Cook A, FitzGerald MG, Swofford R *et al*: **An Improved Canine Genome and a Comprehensive Catalogue of Coding Genes and Non-Coding Transcripts**. *PLoS ONE* 2014, **9**(3):e91172.

52. Gopal SK, Greening DW, Mathias RA, Ji H, Rai A, Chen M, Zhu HJ, Simpson RJ: **YBX1/YB-1 induces partial EMT and tumourigenicity through secretion of angiogenic factors into the extracellular microenvironment**. *Oncotarget* 2015, **6**(15):13718-13730.

53. Hekman JP, Johnson JL, Edwards W, Vladimirova AV, Gulevich RG, Ford AL, Kharlamova AV, Herbeck Y, Acland GM, Raetzman LT *et al*: **Anterior Pituitary Transcriptome Suggests Differences in ACTH Release in Tame and Aggressive Foxes**. *G3 (Bethesda)* 2018, **8**(3):859-873.

54. Boulay G, Sandoval GJ, Riggi N, Iyer S, Buisson R, Naigles B, Awad ME, Rengarajan S, Volorio A, McBride MJ *et al*: **Cancer-Specific Retargeting of BAF Complexes by a Prion-like Domain**. *Cell* 2017, **171**(1):163-178 e119.

55. Stark JM, Jasin M: **Extensive loss of heterozygosity is suppressed during homologous repair of chromosomal breaks**. *Molecular and cellular biology* 2003, **23**(2):733-743.

56. Niedernhofer LJ, Essers J, Weeda G, Beverloo B, de Wit J, Muijtjens M, Odijk H, Hoeijmakers JH, Kanaar R: **The structure-specific endonuclease Ercc1-Xpf is required for targeted gene replacement in embryonic stem cells**. *The EMBO journal* 2001, **20**(22):6540-6549.

57. Chen WK, Swartz JD, Rush LJ, Alvarez CE: **Mapping DNA structural variation in dogs**. *Genome Res* 2009, **19**(3):500-509.

58. Hendricks WPD, Zismann V, Sivaprakasam K, Legendre C, Poorman K, Tembe W, Kiefer J, Liang W, DeLuca V, Stark M *et al*: **Somatic inactivating <em>PTPRJ</em> mutations and dysregulated pathways identified in canine melanoma by integrated comparative genomic analysis**. *bioRxiv* 2017.

59. Clemente-Vicario F, Alvarez CE, Rowell JL, Roy S, London CA, Kisseberth WC, Lorch G: **Human Genetic Relevance and Potent Antitumor Activity of Heat Shock Protein 90 Inhibition in Canine Lung Adenocarcinoma Cell Lines**. *PLoS One* 2015, **10**(11):e0142007.

60. Braunreiter CL, Hancock JD, Coffin CM, Boucher KM, Lessnick SL: **Expression of EWS-ETS fusions in NIH3T3 cells reveals significant differences to Ewing's sarcoma**. *Cell Cycle* 2006, **5**(23):2753-2759.

61. Riggi N, Knoechel B, Gillespie SM, Rheinbay E, Boulay G, Suva ML, Rossetti NE, Boonseng WE, Oksuz O, Cook EB *et al*: **EWS-FLI1 utilizes divergent chromatin remodeling mechanisms to directly activate or repress enhancer elements in Ewing sarcoma**. *Cancer cell* 2014, **26**(5):668-681.

62. Minas TZ, Surdez D, Javaheri T, Tanaka M, Howarth M, Kang HJ, Han J, Han ZY, Sax B, Kream BE *et al*: **Combined experience of six independent laboratories attempting to create an Ewing sarcoma mouse model**. *Oncotarget* 2017, **8**(21):34141-34163.

63. Tanaka M, Yamazaki Y, Kanno Y, Igarashi K, Aisaki K, Kanno J, Nakamura T: **Ewing's sarcoma precursors are highly enriched in embryonic osteochondrogenic progenitors**. *The Journal of clinical investigation* 2014, **124**(7):3061-3074.

64. Michailidou K, Hall P, Gonzalez-Neira A, Ghoussaini M, Dennis J, Milne RL, Schmidt MK, Chang-Claude J, Bojesen SE, Bolla MK *et al*: **Large-scale genotyping identifies 41 new loci associated with breast cancer risk**. *Nat Genet* 2013, **45**(4):353-361, 361e351-352.

65. Michailidou K, Lindstrom S, Dennis J, Beesley J, Hui S, Kar S, Lemacon A, Soucy P, Glubb D, Rostamianfar A *et al*: **Association analysis identifies 65 new breast cancer risk loci**. *Nature* 2017, **551**(7678):92-94.

66. Wolpin BM, Rizzato C, Kraft P, Kooperberg C, Petersen GM, Wang Z, Arslan AA, Beane-Freeman L, Bracci PM, Buring J *et al*: **Genome-wide association study identifies multiple susceptibility loci for pancreatic cancer**. *Nat Genet* 2014, **46**(9):994-1000.

67. Kedage V, Selvaraj N, Nicholas TR, Budka JA, Plotnik JP, Jerde TJ, Hollenhorst PC: **An Interaction with Ewing's Sarcoma Breakpoint Protein EWS Defines a Specific Oncogenic Mechanism of ETS Factors Rearranged in Prostate Cancer**. *Cell reports* 2016, **17**(5):1289-1301.

68. Meyer MB, Benkusky NA, Sen B, Rubin J, Pike JW: **Epigenetic Plasticity Drives Adipogenic and Osteogenic Differentiation of Marrow-derived Mesenchymal Stem Cells**. *The Journal of biological chemistry* 2016, **291**(34):17829-17847.
