# Supplementary material for: Risk-modeling of dog osteosarcoma genome scans shows individuals with Mendelian-level polygenic risk are common
Source: BMC Genomics. 2019 Mar 19;20:226. doi: 10.1186/s12864-019-5531-6 (PMC6425649; doi:10.1186/s12864-019-5531-6)
Supplement: Supplementary file 4 — Figure S1. Cluster analysis of frequencies of canine osteosarcoma GWAS risk alleles across breeds. Figure S2. EWSR1 and canine EWSR1 retrogenes multiple sequence alignment across diverse representative species. Figure S3. Alignment of ZF-RanBP domain and C-terminal Nuclear Localization Signal of EWSR1 and canine EWSR1 retrogenes with those of diverse other species, and with those of the other human proteins with the ZF-RanBP domain. (PDF 940 kb) [file 12864_2019_5531_MOESM4_ESM.pdf]

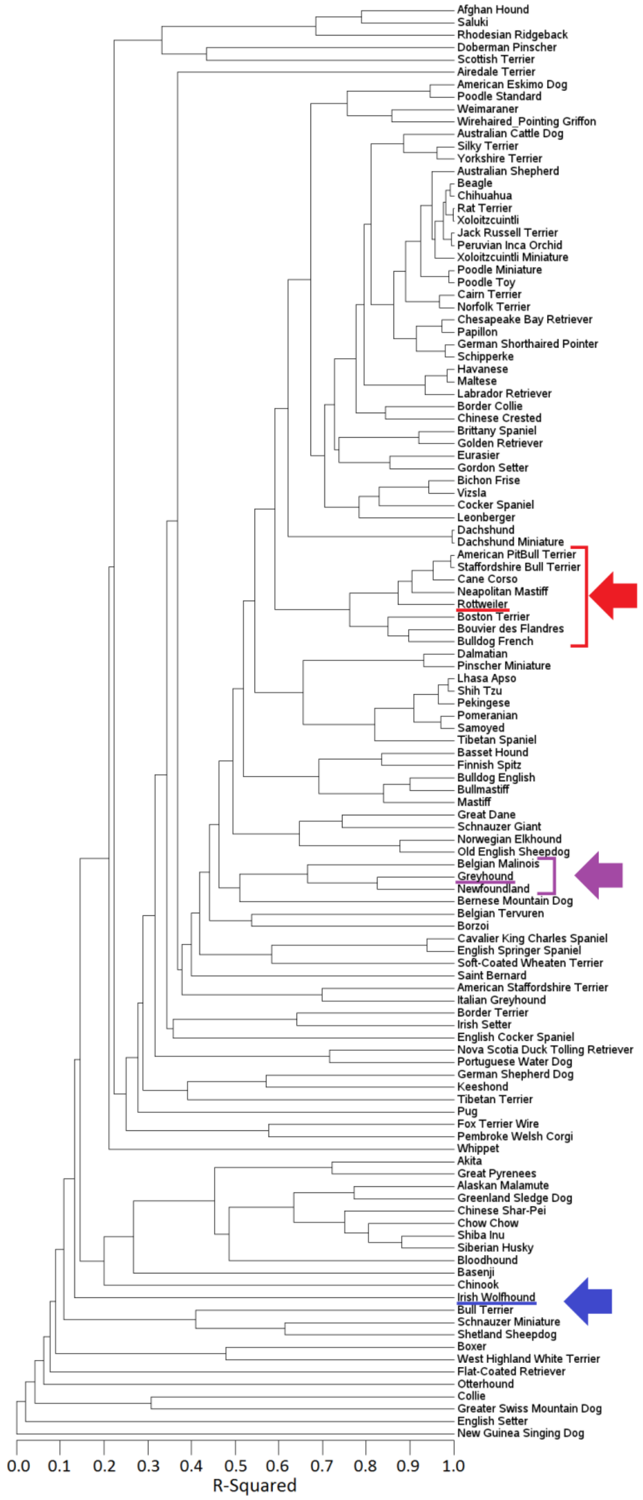

Supplementary Figure S1. Hierarchical cluster tree constructed from allele frequencies from the selected Osteosarcoma GWAS markers and IUT-GWAS marker. Breeds included in the study are underlined and identified by an arrow: Greyhound=Purple, Rottweiler=Red, and Irish Wolfhound =Blue. Correlated breeds are indicated by a bracket of the same colors assigned.

```
Zebrafish           1  MASAADYNSYGQAGSQQGYGSYPAQPSQPGYGQGTQQSYGQQQGYGSYSQPADSTYSQTS
Frog                1  MAST-DYSTYSQTGAQQSYSAYTAQPTQ-GYAQTAQATYGQ-QSYGTYAQPTDASYTQAQ
Chicken             1  MAST-DYSTYSQAAAQQGYSAYAPQPAQ-GYAQTTQ-TYGQ-QSYGTYGQPTDASYTQPQ
Platypus           55  MGST-DYSTYSQAAAQQGYSAYAAQPAQ-GYAQTTQ-AYGQ-QSYGAYGQPTDVSYTQAQ
Koala               1  MAST-DYSTYSQAAAQQGYSAYAAQPAQ-GYAQTTQ-AYGQ-QSYGTYGQPTDVSYTQAQ
Armadillo           1  MAST-DYSTYSQAAAQQGYSAYTAQPAQ-GYAQTTQ-AYGQ-QSYGTYGQPTDVSYTQAQ
Elephant            1  MAST-DYSTYSQAAAQQGYSAYTAQPTQ-GYAQTTQ-AYGQ-QSYGTYGQPTDVSYTQAQ
Big brown bat       1  MAST-DYSTYSQAAAQQGYSAYTAQPTQ-GYAQTTQ-AYGQ-QSYGTYGQPTDVSYTQAQ
Yangtze dolphin     1  MAST-DYSTYSQAAAQQGYSAYTAQPTQ-GYAQTTQ-AYGQ-QSYGTYGQPTDVSYTQAQ
Cow                 1  MAST-DYSTYSQAAAQQGYSAYTAQPTQ-GYAQTTQ-AYGQ-QSYGTYGQPTDVSYTQAQ
Panda               1  MAST-DYSTYSQAAAQQGYSAYTAQPTQ-GYAQTTQ-AYGQ-KSYGTYGQPTDVSYTQAQ
Ferret              1  MAST-DYSTYSQAAAQQGYSAYTAQPTQ-GYAQTTQ-AYGQ-QSYGTYGQPTDVSYTQAQ
Cat                 1  MAST-DYSTYSQAAAQQGYSAYTAQPTQ-GYAQTTQ-AYGQ-QSYGTYGQPTDVSYTQAQ
Dog                 1  MAST-DYSTYSQAAAQQGYSAYTAQPTQ-GYAQTTQ-AYGQ-QSYGTYGQPTDVSYTQAQ
Dog retrogene 1     1  MAST-DYSTYSQAAAQQGYSAYTAQPTQ-GYAQTTQ-AYGQ-QSDGTYGQPTDVSYAQAQ
Dog retrogene 2     1  MVST-DYSIYSQTAAQQGYSAYTTQPTQ-GYAQATQ-AYGQ-QSYGTYGQPTDVSYTQAQ
European shrew     27  MPRR-YYSTYSQAAAQQGYSAYTAQPTQ-GYAQTSQ-AYGQ-QSYGTYGQPTDVSYTQAQ
Rat                 1  MAST-DYSTYSQAAAQQGYSAYTAQPTQ-GYAQTTQ-AYGQ-QSYGTYGQPTDVSYTQAQ
Human               1  MAST-DYSTYSQAAAQQGYSAYTAQPTQ-GYAQTTQ-AYGQ-QSYGTYGQPTDVSYTQAQ

Zebrafish          61  GSSSSYGQQPYGSSYGQQPPASGSYN--AASATPQGYTQPVQGYGSSSYDSSTAASSTS
Frog               58  -TTASYGQSAYATSYGQPPT------GYTAPAAPQAYSQPMQGYGSTGYDSTTATT---T
Chicken            57  -TTATYGQTAYATSYGQPPTVEGTSTGYTAPTAPPAYSQPVQGYGTAAYDTSTATV---T
Platypus           57  -TTATYGQTAYATSYGQPPT------GYTTPTAPQAYSQPVQGYGTGAYDTTTATV---T
Koala              57  -TTATYGQTAYATSYGQPPT------GYTTPTAPQAYSQPVQGYGTGAYDTTTATV---T
Armadillo          57  -TTATYGQTAYASSYGQPPTGKTGLGEIVGS----QFS----------------NI---T
Elephant           57  -TTATYGQTAYATSYGQPPT------GYTTPAAPQAYSQPVQGYGTGAYDTTTATV---T
Big brown bat      57  -TTATYGQTAYATSYGQPPT------GYTTPAAPQAYSQPVQGYGTGAYDTTTATV---T
Yangtze dolphin    57  -TTATYGQTAYATSYGQPPT------GYSTPTAPQAYSQPVQGYGTGAYDTTTATV---T
Cow                57  -TTATYGQTAYATSYGQPPT------GYSTPAAPQAYSQPVQGYGTGAYDTTTATV---T
Panda              57  -TTATYGQTAYATSYGQPPA------GYTTPTAPQAYSQPVQGYGTGAYDTTTATV---T
Ferret             57  -TTATYGQTAYATSYGQPPA------GYTTPTAPQAYSQPVQGYGTGAYDTTTATV---T
Cat                57  -TTATYGQTAYATSYGQPPAVEGTSTGYTTPTAPQAYSQPVQGYGSGAYDTTTATV---T
Dog                57  -TTATYGQTAYATSYGQPPA------GYTTPTAPQAYSQPVQGYGTGAYDTTTATV---T
Dog retrogene 1    57  -TTATYGQTAYATSYGQPPA------GYTTPTAPQAYSQPVQGYGTGAYDTTTATV---T
Dog retrogene 2    57  -TTATYGQTAYATSYGQPPA------GYTTPTAP-AYSQPVQWYDTGAYDTTTAMV---T
European shrew     57  -TTATYGQTAYATSYGQPPTVEGTNTGYTTPTAPQAYSQPVQGYGTGAYDTTTATV---T
Rat                57  -STATYGQTAYATSYGQPPT------GYTTPTAPQAYSQPVQGYGTGAYDTTTATV---T
Human              57  -TTATYGQTAYATSYGQPPTVEGTSTGYTTPTAPQAYSQPVQGYGTGAYDTTTATV---T
                                      aaaaaaaaaaaaaaaaaaaaaaaaaaaaaaaaaaaaaaaaaaaaa

Zebrafish         119  NTQTSYGGQASYGTQSAYPGYGQQPAAAAPPSSYS---------------S---------
Frog              108  TTQATYAAPSAYGSQPAYPSYGQQPATTASARPQDGSKPADTSQPPPSTASYAQPSMGYS
Chicken           107  TTQASYAAPSAYGTQPAYPAYGQQPPAAVPPRPQDGSKPAETSQPPSSTTGYNQPSLGYG
Platypus          107  TTQASYAAQSAYGTQPAYPTYGQQPAATAPTRPQDGSKPAETSQPQSSTAGYSQPSLGYG
Koala             107  TTQASYAAQSAYGTQPAYPTYGQQPAAAAPARPQDGSKPAETSQPQSSTAGYSQPSLGYG
Armadillo          93  ------------HDKPRVLWLRNEVFFISPFRPQDGSKPAETSQPQSSTGGYNQPSLGYG
Elephant          107  TTQASYAAQSAYGTQPAYPAYGQQPAATAPARPQDGSKPTETSQPQSSTGGYNQPSMGYG
Big brown bat     107  TTQASYAAQSAYGTQPAYPAYGQQPATTAPARPQDGNKPAETSQPQSSTGGYNQPSLGYG
Yangtze dolphin   107  TTQASYAAQSAYGTQPAYPAYGQQPAATAPARPQDGNKPAETSQPQSSTGGYNQPSLGYG
Cow               107  TTQASYAAQSAYGTQPAYPAYGQQPAATAPARPQDGNKPAETSQPQSSTGGYNQPSLGYG
Panda             107  TTQVSYAAQSACGTQPVYPAYGQQPAATAPARPQDGNKPAETSQPQSSTGGYNQPSLGYG
Ferret            107  TTQASYAAQSAYGTQPAYPAYGQQPAATAPARPQDGNKPAETSQPQSSTGGYNQPSLGYG
Cat               113  TTQASYAAQSAYGTQPAYPAYGQQPAATAPARPQDGNKPAETSQPQSSTGGYNQPSLGYG
Dog               107  TTQASYAAQSAYGTQPAYPAYGQQPAATAPARPQDGNKPAETSQPQSSTGGYNQPSLGYG
Dog retrogene 1   107  TTQASYAAQSAYGTQPAYPAYGQQPAATAPARPQDGNKPTETSQPQSSTGGYNQPSLGYG
Dog retrogene 2   106  STQASYAAQSAYGTQPAYPACGLQPAATAPARPQDGNKPAETSQPQSSTGGYNQPSLGYG
European shrew    113  TTQASYAAQSAYGTQPAYPAYGQQPATTAPTRPQDGNKPAETSQPQSSTGGYNQPSLGYG
Rat               107  TTQASYAAQSAYGTQPAYPAYGQQPAATAPTRPQDGNKPAETSQPQSSTGGYNQPSLGYG
Human             113  TTQASYAAQSAYGTQPAYPAYGQQPAATAPTRPQDGNKPTETSQPQSSTGGYNQPSLGYG
                       aaaaaaaaaaaaaaaaaaaaaaaaaaaaaaaaaaaaaaaaaa
```

```
Zebrafish          155 -----------------------------GSQQPAGYEQSSYSQQPQQSTYSQQQQGGYQ
Frog               168 QSSYSYPQVPASYPMQQVSAPPSYPPTSYSSSQTSSYEQSSYSQPSSYTQQSGYGQQASY
Chicken            167 QSNYSYPQVPASYPMQPVTAPPSYPPTSYSSTQPSSYDQSTYSQQST-------------
Platypus           167 QSNYSYPQVPSSYPMQPVTAPPSYPPTSYSSTQPTSYDQSTYSQQNT-------------
Koala              167 QSNYSYPQVPGSYPMQPVTAPPSYPPTSYSSTQPTSYDQSSYSQQNT-------------
Armadillo          141 QSNYSYPQVPGSYPMQPVTAPPSYPPTSYSSSQPTSYDQSSYSQQNT-------------
Elephant           167 QSNYSYPQVPGSYPMQPVTAPPSYPPTSYSSSQPTSYDQSSYSQQNT-------------
Big brown bat      167 QSNYSYPQVPGSYPMQPVTAPPSYPPTSYSSSQPTSYDQSSYSQQNT-------------
Yangtze dolphin    167 QSNYSYPQVPGSYPMQPVTAPPSYPPTSYSSTQPTSYDQSSYSQQNT-------------
Cow                167 QSNYSYPQVPGSYPMQPVSAPPSYPPTSYSSTQPTSYDQSSYSQQNT-------------
Panda              167 QSNYSYPQVPGSYPMQPVTAPPSYPPTSYSSTQPTSYDQSSYSQQNT-------------
Ferret             167 QSNYSYPQVPGSYPMQPVTAPPSYPPTSYSSTQPTSYDQSSYSQQNT-------------
Cat                173 QSNYSYPQVPGSYPMQPVTAPPSYPPTSYSSTQPTSYDQSSYSQQNT-------------
Dog                167 QSNYSYPQVPGSYPMQPVTAPPSYPPTSYSSTQPTSYDQSSYSQQNT-------------
Dog retrogene 1    167 QSNYSYPWGPGSYLMQPVTAPPLYPPTSYSSTQPTSYDKSSYSQQNT-------------
Dog retrogene 2    166 QSNYSYTQVPGSYRMQPVTAPPSYPPTSYSSTQPTSYDQSSYLQQNT-------------
European shrew     173 QSNYSYPQVPGSYPMQPVTAPPSYPPTSYSSTQPTSYDQSSYSQQNT-------------
Rat                167 QSNYSYPQVPGSYPMQPVTAPPSYPPTSYSSSQPTSYDQSSYSQQNT-------------
Human              173 QSNYSYPQVPGSYPMQPVTAPPSYPPTSYSSTQPTSYDQSSYSQQNT-------------

Zebrafish          186 GQQGGYGQQSSYSQQGGYQ-----QTPPQQQQAPPSSYAPPSGSYGQPSASQYGQQGSTG
Frog               228 SQQSSYGQPSSYGQQSGYGQQSSYQAPPQQQQPPPTSYPPPSSSYSSQPPGQYGQQSSSY
Chicken            214 -----YGQQNTYSQQTSYGQQSS-----YAQQPPPTSYPPQTGSYS-QPPSQYSQQSSSY
Platypus           214 -----YGQQSSYGQPSSYGQQSS------YGQQPPTSYPPPTGSYS-QAPSQYSQQSSSY
Koala              214 -----YGQQSSYGQQSSYGQQSS------YGQQPPTSYPPQTGSYS-QAPSQYSQQSSSY
Armadillo          188 -----YGQPSSYGQQSSYGQQSS------YGQQPPTSYPPQTGSYS-QAPSQYSQQSSSY
Elephant           214 -----YGQPSSYGQQSSYGQQSS------YGQQPPTSYPPQTGSYS-QAPSQYSQQSSSY
Big brown bat      214 -----YGQPSSYGQQSSYGQQKQSS----YGQQPPTSYPPQTGSYS-QAPSQYSQQSSSY
Yangtze dolphin    214 -----YGQPSSYGQQSSYGQQSS------YGQQPPTSYPPQTGSYS-QAPSQYSQQSSSY
Cow                214 -----YGQPSSYGQQSSYGQQSS------YGQQPPTSYPPQTGSYS-QAPSQYSQQSSSY
Panda              214 -----YGQPSSYGQQSSYGQQSS------YGQQPPTSYPPQTGSYS-QAPSQYSQQSSSY
Ferret             214 -----YGQPSSYGQQSSYGQQSS------YGQQPPTSYPPQTGSYS-QAPSQYSQQSSSY
Cat                220 -----YGQPSSYGQQSSYGQQSS------YGQQPPTSYPPQTGSYS-QAPSQYSQQSSSY
Dog                214 -----YGQPSSYGQQSSYGQQSS------YGQQPPTSYPPQTGSYS-QAPSQYSQQSSSY
Dog retrogene 1    214 -----YGQRSSYGQQSSYGQQSS------YGQQLPTSYPPQTGSYS-QAPSQYSQQSSSY
Dog retrogene 2    213 -----YGQPSSY------------------------------------------------
European shrew     220 -----YGQPSSYGQQSSYGQQSS------YGQQPPTSYPPQTGSYS-QAPSQYSQQSSSY
Rat                214 -----YGQPSSYGQQSSYGQQSS------YGQQPPTSYPPQTGSYS-QAPSQYSQQSSSY
Human              220 -----YGQPSSYGQQSSYGQQSS------YGQQPPTSYPPQTGSYS-QAPSQYSQQSSSY

Zebrafish          241 GGYGQSDYKPPNQYGSYRPDHQNGGGYSGPES-GGYGGPGEGRGMGGGENRGRGRGGFDR
Frog               288 G-----------QQSSYRPDHSSSKSPYGQEPHQGFSSSGENRGSGGSDSRSRGRGMFER
Chicken            263 G-----------QQSSFRQDHPSSMNVYGQES-GGFSGPGENRNMSGPDNRGRGRGGYER
Platypus           262 G-----------QQSAFRQDHPSSMGVYGQES-GGFSGPGESRSMSGPDNRGRGRGGFDR
Koala              262 G-----------QQSAFRQDHPSSMGVYGQES-GGFSGPGENRSMSGPDNRGRGRGGFDR
Armadillo          236 G-----------QQSSFRQDHPSSMGVYGQES-GGFSGPGENRSMSGPDNRGRGRGGFDR
Elephant           262 G-----------QQSSFRQDHPSSMGVYGQES-GGFSGPGENRSMSGPDNRGRGRGGFDR
Big brown bat      264 G-----------QQIYLGGL-------------------------------GRTKNGFEI
Yangtze dolphin    262 G-----------QQSSFRQDHPSSMGVYGQES-GGFSGPGENRSMSGPDNRGRGRGGFDR
Cow                262 G-----------QQSSFRQDHPSSMGVYGQES-GGFSGPGENRSMSGPDNRGRGRGGFDR
Panda              262 G-----------QQSSFRQDHPSSMGVYGQES-GGFSGPGENRSMSGPDNRGRGRGGFDR
Ferret             262 G-----------QQSSFRQDHPSSMGVYGQES-GGFSGPGENRSMSGPDNRGRGRGGFDR
Cat                268 G-----------QQSSFRQDHPSSMGVYGQES-GGFSGPGENRSMSGPDNRGRGRGGFDR
Dog                262 G-----------QQSSFRQDHPSSMGVYGQES-GGFSGPGENRSMSGPDNRGRGRGGFDR
Dog retrogene 1    262 G-----------QQSSFRQYHPSSMGVYGQES-GGFSGPGENQSMSGLENRGRGRGGFDC
Dog retrogene 2    220 ----------------------------GQES-GGFSGPGENWSMSGPDNRGRGRGGFNR
European shrew     268 G-----------QQSSFRQDHPSSMGVYGQES-GGFSGPGENRSMSGPDNRGRGRGGFDR
Rat                262 G-----------QQSSFRQDHPSSMGVYGQES-GGFSGPGENRSLSGPDNRGRGRGGFDR
Human              268 G-----------QQSSFRQDHPSSMGVYGQES-GGFSGPGENRSMSGPDNRGRGRGGFDR
```

```
Zebrafish        300 GMMRGGGGMRGGMSRGGMGIAGDRGGFSKPGDGEMG--------AP-------EEQDDSEN
Frog             337 GGMSRGGRG----SRGGMGSGGDRAGFSKPGGLLDDGPDLDLGPPMPLPLPLDLEDELES
Chicken          311 GGMSRGGRG---GGRGGMG-AGERGGFNKPGGHMEEGPDLDLGPPM------DPDEDSDN
Platypus         310 GGMSRGGRG---GGRGGMGSAGERGGFNKPGGPMEEGPDLDLGPPV------DPDEDSEN
Koala            310 GGMSRGGRGGGGGRGGMGSAGERGGFNKPGGPMEEGPDLDLGPPV------DPDEDSEN
Armadillo        284 GGMSRGGRGG---GRGGMGSAGERGGFNKPGGPMDEGPDLDLGPPV------DPDEDSDN
Elephant         310 GGMSRGGRG---GGRGGMGSAGERGGFNKPGGPMDEGPDLDLGPPV------DPDEDSDN
Big brown bat    282 GAF---------SSLHGFQSAGERGGFNKPGGPMDEGPDLDLGPPV------DPDEDSDN
Yangtze dolphin  310 GGMSRGGRG---GGRGGMGSAGERGGFNKPGGPMDEGPDLDLGPPV------DPDEDCDN
Cow              310 GGMSRGGRG---GGRGGMG-AGERGGFNKPGGPMDEGPDLDLGPPV------DPDEDSDN
Panda            310 GGMSRGGRG---GGRGGMGSAGERGGFNKPGGPMDEGPDLDLGPPV------DPDEDCDN
Ferret           310 GGMSRGGRG---GGRGGMGSAGERGGFNKPGGPMDEGPDLDLGPPV------DPDEDSDN
Cat              316 GGMSRGGRG---GGRGGMGSAGERGGFNKPGGPMDEGPDLDLGPPV------DPDEDSDN
Dog              310 GGMSRGGRG---GGRGGMGSAGERGGFNKPGGPMDEGPDLDLGPPV------DPDEDSDN
Dog retrogene 1  310 GGISGGGWG---GGLGGMG-AGERGGFNKPGGSMDEGPALNLGPPV------DPDEDSDN
Dog retrogene 2  251 GGMSRGGPG---GGRGGMGSAGERGGFNKPGGPMDEGPDLDLGPPV------DPDEDSDN
European shrew   316 GGMSRGGRG---GGRGGMG-AGERGGFNKPGGPMDEGPDLDLGPPI------DPDEDSDN
Rat              310 GGMSRGGRG---GGRGGMGSAGERGGFNKPGGPMDEGPDLDLGLPI------DPDEDSDN
Human            316 GGMSRGGRG---GGRGGMG-AGERGGFNKPGGPMDEGPDLDLGPPV------DPDEDSDN

Zebrafish        346 STIYITGLTENATLEEVADFFKHSGIIRINKRTGLPAVNIYTDKDTGKPKGDATLSYEEP
Frog             393 STIYVQGLNDNVTVEEIVDFFKHCGDVKINKRTGEPLVNLFMDKETGKPKGDGTVSFEDP
Chicken          362 SSVYVQGLNDNVTLEDLADFFKQCGVVKMNKRTGQPMIHLYIDKETGKPKGDATVSYDDP
Platypus         361 TAIYVQGLNENVTLEDLADFFKQCGVLKMNKRTGQPLINIYLDKETGKPKGDATVAYDDS
Koala            364 SAIYVQGLNENVTVDELADFFKQCGVVKMNKRTGQPMINIYLDKETGKPKGDATVSYDDP
Armadillo        335 TAIYVQGLNDNVTLDDLADFFKQCGVVKMNKRTGQPMIHIYLDKETGKPKGDATVSYEDP
Elephant         361 SAIYVQGLNDSVTLDDLTDFFKQCGVVKMNKRTGQPMIHIYLDKETGKPKGDATVSYEDP
Big brown bat    327 SAIYVQGLNDNVTLDDLADFFKQCGVVKMNKRTGQPMIHIYLDKETGKPKGDATVSYEDP
Yangtze dolphin  361 SAIYVQGLSDNVTLDDLADFFKQCGVVKMNKRTGQPMIHIYLDKETGKPKGDATVSYEDP
Cow              360 SAIYVQGLNDSVTLDDLVDFFKQCGVVKMNKRTGQPMIHIYLDKETGKPKGDATVSYEDP
Panda            361 SAIYVQGLNDSVTLDDLADFFKQCGVVRMNKRTGQPMIHIYLDKETGKPKGDATVSYEDP
Ferret           361 SAIYVQGLNDNVTLDDLADFFKQCGVVKMNKRTGQPMIHIYLDKETGKPKGDATVSYEDP
Cat              367 SAIYVQGLNDNVTLDDLADFFKQCGVVKMNKRTGQPMIHIYLDKETGKPKGDATVSYEDP
Dog              361 SAIYVQGLNDNVTLDDLADFFKQCGVVKMNKRTGQPMIHIYLDKETGKPKGDATVSYEDP
Dog retrogene 1  364 RAIYVQGLNDNVTLDDLADFFKQCGVNKMNKRTGQPMIHIYLDKERGKPKGDATVSYEDP
Dog retrogene 2  302 SAIYVQGLNDNVTLDDLADFFKQCGLVKMNKRTGQPMIHIYLDKETGKPKGDATVSYEDP
European shrew   366 SAIYVQGLNDNVTLDDLADFFKQCGVVKMNKRTGQPMIHIYLDKETGKPKGDATVSYEDP
Rat              361 SAVYVQGLNDSVTLDDLADFFKQCGVVKMNKRTGQPMIHIYLDKETGKPKGDATVSYEDP
Human            366 SAIYVQGLNDSVTLDDLADFFKQCGVVKMNKRTGQPMIHIYLDKETGKPKGDATVSYEDP
                     rrrrrrrrrrrrrrrrrrrrrrrrrrrrrrrrrrrrrrrrrrrrrrrrrrrrrrrrrrrr

Zebrafish        406 PSAKAAVEWFDGKDFQGKKLKVSMARRKPMMGMMRGGMPMRGDRGGMM------------
Frog             453 PSAKTAIELCDGKDLNGNKVKVSLARKKSLLGSMRGGSLLRDNRGQPPPLRG--------
Chicken          422 STAKTAVEWFDGKDFQGSKLKVSLTRKKGPMNSMRGGMPPRDQRGMPPPLRGGPGGPGGP
Platypus         421 PTAKAAVEWFDGKDFQGSKLKVSLARKKPPMNSLRGGMPPREGRGMPPPLRGGPGGPGG-
Koala            424 PTAKAAVEWFDGKDFQGSKLKVSLARKKPPMNSMRGGMPPREGRGMPPPLRGGPGGPGG-
Armadillo        395 PTAKAAVEWFDGKDFQGSKLKVSLARKKPPMNSMRGGMPPREGRGMPPPLRGGPGGPGG-
Elephant         421 PTAKAAVEWFDGKDFQGSKLKVSLARKKPPMNSMRGGMPPREGRGMPPPLRGGPGGPGG-
Big brown bat    387 PTAKAAVDWFDGKDFQGSKLKVSLARKKPPMNSMRGGMPPREGRGMPPPLRGGPGGPGG-
Yangtze dolphin  421 PTAKTAVEWFDGKDFQGSKLKVSLARKKPPMNSMRGGMPPREGRGMPPPLRGGPGGPGG-
Cow              420 ATAKAAVEWFDGKDFQGSKLKVSLARKKPPMNSMRGGMPPREGRGMPPPLRGGPGGPGG-
Panda            421 PTAKAAVEWFDGKDFQGSKLKVSFARKKPPMNSMRGAMPPREGRGMPPPLRGGPGGPGG-
Ferret           421 PTAKAAVEWFDGKDFQGSKLKVSLARKKPPMNSLRGGMPPREGRGMPPPLRAGPGGPGG-
Cat              427 PTAKAAVEWFDGKDFQGSKLKVSLARKKPPMNSMRGGMPPREGRGMPPPLRGGPGGPGG-
Dog              421 PTAKAAVEWFDGKDFQGSKLKVSLARKKPPMNSMRGGMPPREGRGMPPPLRGGPGGPGG-
Dog retrogene 1  421 PTAKAAVEWFDGRDFQGSKLKVSLARKKPPMNSMRGRMPSSEGKGVPPPLRGGPGGPGG-
Dog retrogene 2  362 PTAKAAVEWFDGKDFQGSKLKVSLAGKKPPMNSMRGGMPPR------------PGGPGG-
European shrew   426 PTAKAAVEWFDGKDFQGSKLKVSLARKKPPMNSMRGGMPPREGRGMPPPLRGGPGGPGG-
Rat              421 PTAKAAVEWFDGKDFQGSKLKVSLARKKPPLNSMRGGMPPREGRGMPPPLRGGPGGPGG-
Human            426 PTAKAAVEWFDGKDFQGSKLKVSLARKKPPMNSMRGGLPPREGRGMPPPLRGGPGGPGG-
                     rrrrrrrrrrrrrrrrrrrrrrrr
```

```
Zebrafish          454 ---GRGGMMGRGGMGRGGDRGGFMPRGGPRGMGRGGPT-GGNMQQRAGDWQCPNAGCGNQ
Frog               505 ------GPMGRL-GGRGGERGGFMPRG-PRGP-RGSPVS-GNVQHRAGDWQCPNPGCGNQ
Chicken            478GGPGGPSGPMGRM-GGRGGDRGGFSSRG-PRGS-RGNPSG-GSVQHRAGDWQCPNPGCGNQ
Platypus           480 ----PGGPMGRM-GGRGGERGGFPPRG-PRGS-RGNPSGGGNVQHRAGDWQCPNPGCGNQ
Koala              483 ----PGGPMGRM-GGRGGDRGGFPPRG-PRGS-RGNPSGGGNVQHRAGDWQCPNPGCGNQ
Armadillo          454 ----PGGPMGRM-GGRGGERGGFPPRG-PRGS-RGNPSGGGNVQHRAGDWQCPNPGCGNQ
Elephant           480 ----PGGPMGRM-GGRGGDRGGFPPRG-PRGS-RGNPSGGGNVQHRAGDWQCPNPGCGNQ
Big brown bat      446 ----PGGPMGRM-GGRGGDRGGFPPRG-PRGS-RGNPSGGGNVQHRAGDWQCPNPGCGNQ
Yangtze dolphin    480 ----PGGPMGRM-GGRGGDRGGFPPRG-PRGS-RGNPSGGGNVQHRAGDWQCPNPGCGNQ
Cow                479 ----PGGPMGRM-GGRGGDRGGFPPRG-PRGS-RGNPSGGGNVQHRAGDWQCPNPGCGNQ
Panda              480 ----PGGPMGRM-GGRGGDRGGFPPRG-PRGS-RGNPSGGGNVQHRAGDWQCPNPGCGNQ
Ferret             480 ----PGGPMGRM-GGRGGDRGGFPPRG-PRGS-RGNPSGGGNVQHRAGDWQCPNPGCGNQ
Cat                486 ----PGGPMGRM-GGRGGDRGGFPPRG-PRGS-RGNPSGGGNVQHRAGDWQCPNPGCGNQ
Dog                480 ----PGGPMGRM-GGRGGDRGGFPPRG-PRGS-RGNPSGGGNVQHRAGDWQCPNPGCGNQ
Dog retrogene 1    480 ----PGGPMGRM-GGRGGDRGGFPPRG-PRVPGRKRPSGGGNFQHRTGDWQCPIPGCGNQ
Dog retrogene 2    409 ----PGGPMGRM-GGRGGDRGGFPPGG-PRGS-RGNPSG--SVQHRAGDWQCPNPGCGNQ
European shrew     485 ----PGGPMGRM-GGRGGDRGGFPPRG-PRGS-RGNPSGGGNVQHRAGDWQCPNPGCGNQ
Rat                480 ----PGGPMGRM-GGRGGDRGGFPPRG-PRGS-RGNPSGGGNVQHRAGDWQCPNPGCGNQ
Human              485 ----PGGPMGRM-GGRGGDRGGFPPRG-PRGS-RGNPSGGGNVQHRAGDWQCPNPGCGNQ
                                                                    zzzzzzzzzzzzzzz

Zebrafish          510 NFAWRMECNQCKAPKPEGFGPPPFPPGGD-RGRGGPGGMRGGRG---MDRGGPGGPGGFR
Frog               555 NFAWRTECNQCKAPKPDGPFPP-FPPQGGERGRGGP-GMRGGRG--LMDRGVPG---MFR
Chicken            544 NFAWRTECNQCKAPKPEGFLPPPFPPPGGDRGRGGPGGMRGGRGGGMMERGGPGG--MFR
Platypus           533 NFAWRTECNQCKAPKPEGFLPPPFPPPGSDRGRGGPGGMRGGRGG-LMDRGGPGG--MFR
Koala              536 NFAWRTECNQCKAPKPEGFLPPPFPPPGGDRGRGGPGGMRGGRGG-LMDRGGPGG--MFR
Armadillo          507 NFAWRTECNQCKAPKPEGFLPPPFPPPGGDRGRGGPGGMRGGRGG-LMDRGGPGG--MFR
Elephant           533 NFAWRTECNQCKAPKPEGFLPPPFPPPGGDRGRGGPGGMRGGRGG-LMDRGGPGG--MFR
Big brown bat      499 NFAWRTECNQCKAPKPEGFLPPPFPPPGGDRGRGGPGGMRGGRGG-LMDRGGPGG--MFR
Yangtze dolphin    533 NFAWRTECNQCKAPKPEGFLPPPFPPPGGDRGRGGPGGMRGGRGG-LMDRGGPGG--MFR
Cow                532 NFAWRTECNQCKAPKPEGFLPPPFPPPGGDRGRGGPGGMRGGRGG-LMDRGGPGG--MFR
Panda              533 NFAWRTECNQCKAPKPEGFLPPPFPPPGGDRGRGGPGGMRGGRGG-LMDRGGPGG--MFR
Ferret             533 NFAWRTECNQCKAPKPEGFLPPPFPPPGGDRGRGGPGGMRGGRGG-LMDRGGPGG--MFR
Cat                539 NFAWRTECNQCKAPKPEGFLPPPFPPPGGDRGRGGPGGMRGGRGG-LMDRGGPGG--MFR
Dog                533 NFAWRTECNQCKAPKPEGFLPPPFPPPGGDRGRGGPGGMRGGRGG-LMDRGGPGG--MFR
Dog retrogene 1    534 NFAWRTECNQCKASKPEGFLPPPFPPPGSDRGRGGPGGMRGGRGG-LMDRGALGG-GMFR
Dog retrogene 2    460 NFAWRTERNQGKAPRPEGFFQHPSRARGP--WQRRPGGLRGGRGG-LRDRGGPGG--MFR
European shrew     538 NFAWRTECNQCKAPKPEGFLPPPFPPPGGDRGRGGPGGMRGGRGG-LMDRGGPGG--MFR
Rat                533 NFAWRTECNQCKAPKPEGFLPPPFPPPGGDRGRGGPGGMRGGRGG-LMDRGGPGG--MFR
Human              538 NFAWRTECNQCKAPKPEGFLPPPFPPPGGDRGRGGPGGMRGGRGG-LMDRGGPGG--MFR
                       zzzzzzzzzzzzzzz

Zebrafish          566 GGRGVDRGGFRG-RGMDRGGFGGRGR-GGPP-------MDDM-GR-R-GRGMGPPGKMDMK
Frog               609 GGRGGDRGGFRG-RGMDR-GFGGG-RRGGPPGPP-GPLMEQLGGRIGGGRR-GGPSKMD-K
Chicken            602 GGRGGDRGGFRGGRGMDRGGYGGGGRRGGGPGGP-PGPLMDQMGGGGRGGRRGGPGKMD-K
Platypus           590 GGRGGDRGGFRGGRGMDRGGFGGG-RRGGPGGPP-GPLMEQMGGR-R-GR--GGPGKMD-K
Koala              593 GGRGGDRGGFRGGRGMDRGGFGGG-RRGGPGGPP-GPLMEQMGGR-RGGR--GGPGKMD-K
Armadillo          564 GGRGGDRGGFRGGRGMDRGGFGGG-RRGGPGGPP-GPLMEQMGGR-RGGR--GGPGKMD-K
Elephant           590 GGRGGDRGGFRGGRGMDRGGFGGG-RRGGPGGPP-GPLMEQMGGR-RGGR--GGPGKMD-K
Big brown bat      556 GGRGGDRGGFRGGRGMDRGGFGGG-RRGGPGGPP-GPLMEQMGGR-RGGR--GGPGKMD-K
Yangtze dolphin    590 GGRGGDRGGFRGTRGMDRGGFGGG-RRGGPGGPP-GPLMEQMGGR-RGGR--GGPGKMD-K
Cow                591 GGRGGDRGGFRGGRGMDRGGFGGG-RRGGPGGPP-GPLMEQMGGR-RGGR--GGPGKMD-K
Panda              590 GGRGGDRGGFRGGRGMDRGGFGGG-RRGGPGGPP-GPLMEQMGGR-RGGR--GGPGKMD-K
Ferret             590 GGRGGDRGGFRGGRGMDRGGFGGG-RRGGPGGPP-GPLMEQMGGR-RGGR--GGPGKMD-K
Cat                596 GGRGGDRGGFRGGRGMDRGGFGGG-RRGGPGGPP-GPLMEQMGGR-RGGR--GGPGKMD-K
Dog                590 GGRGGDRGGFRGGRGMDRGGFGGG-RRGGPGGPP-GPLMEQMGGR-RGGR--GGPGKMD-K
Dog retrogene 1    591 GGRGGDRGGFRGGRGMDRGGFGRG-RRGGPGGPPPGPLMEQMGGR-RGGR--GGPGKMD-K
Dog retrogene 2    515 GGRGGDRGGFRGGRGMAPGGLGGG-RRGVPVGPL-AL-GWNRGRK-KRRR--GGPGKLD-K
European shrew     595 GGRGGDRGGFRGGRGMDRGGFGGG-RRGGPGGPP-GPLMEQMGGR-RGGR--GGPGKMD-K
Rat                590 GGRGGDRGGFRGGRGMDRGGFGGG-RRGGPGGPP-GPLMEQMGGR-RGGR--GGPGKMD-K
Human              595 GGRGGDRGGFRGGRGMDRGGFGGG-RRGGPGGPP-GPLMEQMGGR-RGGR--GGPGKMD-K
```

```
Zebrafish          615 GDHRQDRRERPY
Frog               663 SEHRQERRERPY
Chicken            661 GEHRQERRDRPY
Platypus           644 GEHRQERRDRPY
Koala              648 GEHRQERRDRPY
Armadillo          618 GEHRQERRDRPY
Elephant           645 GEHRQERRDRPY
Big brown bat      611 GEHRQERRDRPY
Yangtze dolphin    645 GEHRQERRDRPY
Cow                653 GEHRQERRDRPY
Panda              645 GEHRQERRDRPY
Ferret             645 GEHRQERRDRPY
Cat                651 GEHRQERRDRPY
Dog                645 GEHRQERRDRPY
Dog retrogene 1    647 GEHRQEHRDWPC
Dog retrogene 2    569 GEHRQGRRDRPC
European shrew     650 GEHRQERRDRPY
Rat                645 GEHRQERRDRPY
Human              650 GEHRQERRDRPY
                         nnnnnnnnnn
```

Supplementary Figure S2. EWSR1 canine retrogene: multiple protein sequence alignment of dog retrogene and the parent EWSR1 gene from diverse representative vertebrates. Three carnivores mentioned in the main article are also included. Alignment was performed using Clustal Omega (EMBL-EBI [1]) and displayed with BoxShade. Black highlighting represents consensus sites, conservative amino acid differences are grey, and non-consensus sites in EWSR1 canine retrogene are yellow. Protein domains were determined using the ancestral EWSR1 protein from dog and the NCBI Domain Search algorithm; and are shown as a single letter abbreviation in blue highlighting below the alignment: Atrophin-1 super family (p=1.4E-4); zf-RanBP (p=5.3E-10); and RRM_EWS (p=2.5E-54). The Nuclear Localization Signal motif (aka, PY motif) is shown in pink [2]. Species names and GenBank accession numbers follow: Dotg retrogene 1 [Canis lupus familiaris, ENSCAFG00000012292.2, translation of transcript ENSCAFT00000019542.2], Dog retrogene 2 [Canis lupus familiaris, ENSCAFG00000000749, translation of transcript ENSCAFT00000001155.3], Dog [Canis lupus familiaris, XP_865186.1], Cat [Felis catus, XP_019670384.1], Panda [Ailuropoda melanoleuca, XP_002915634.1], Ferret [Mustela putorius furo, XP_004766556.1], Human [Homo sapiens, NP_053733.2], Rat [Rattus norvegicus, XP_003749731.1], Cow [Bos Taurus, NP_001103270.1], Koala [Phascolarctos cinereus, XP_020852013.1], Big brown bat [Eptesicus fuscus, XP_008140693.1], Yangtze River dolphin [Lipotes vexillifer, XP_007461224.1], European shrew [Sorex araneus, XP_004615678.1], Elephant [Loxodonta Africana, XP_010597062.1], Armadillo [Dasypus novemcinctus, XP_023445520.1], Platypus [Ornithorhynchus anatinus, XP_001516464.2], Chicken [Gallus gallus, XP_015150340.1], Frog [Xenopus tropicalis, NP_989309.1], Zebrafish [Danio rerio, NP_001108610.1]

Reference

1. Sievers F, Wilm A, Dineen DG, Gibson TJ, Karplus K, Li W, Lopez R, McWilliam H, Remmert M, Söding J, Thompson JD, Higgins DG (2011). Fast, scalable generation of high-quality protein multiple sequence alignments using Clustal Omega. *Molecular Systems Biology* **7**:539 doi:10.1038/msb.2011.75
2. Zakaryan, R.P., Gehring, H. Identification and characterization of the nuclear localization/retention signal in the EWS proto-oncoprotein, J Mol Biol 363 (2006) 27–38.

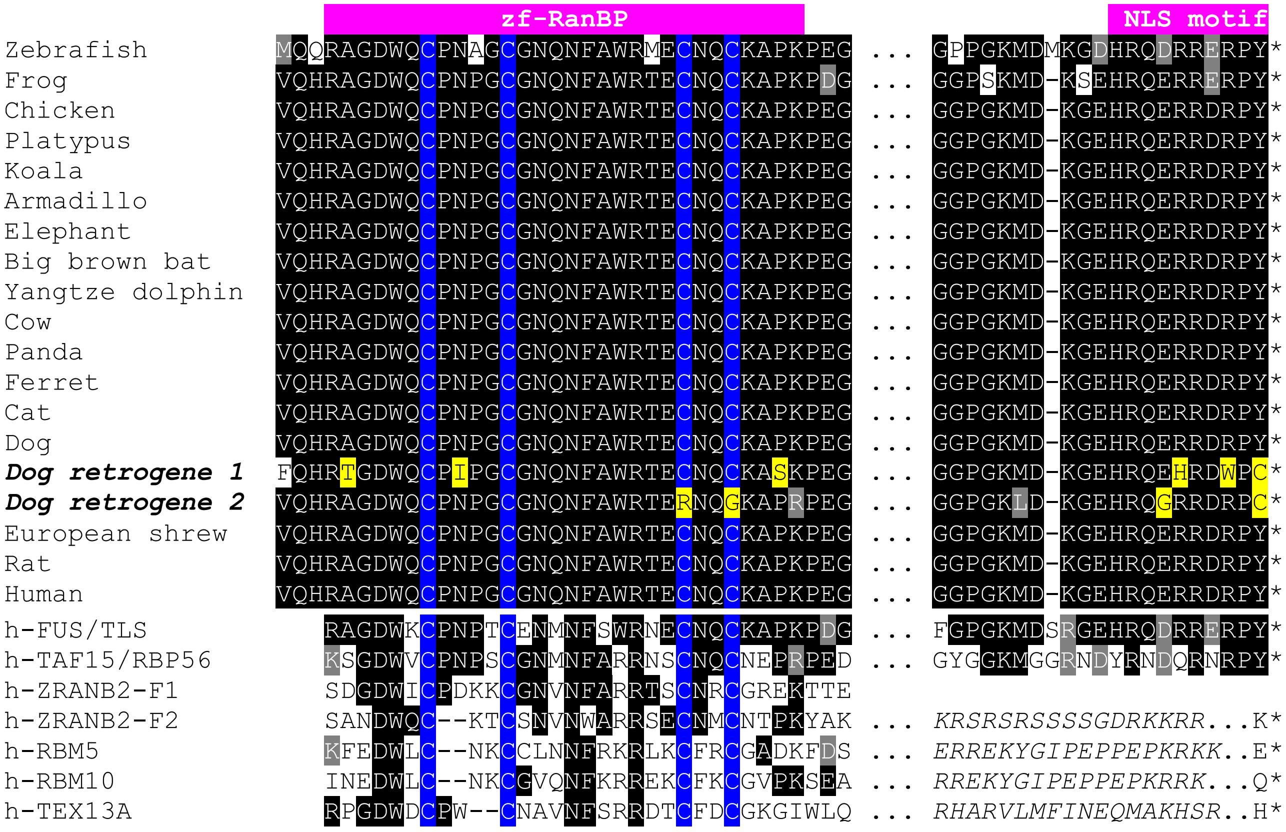

Supplementary Figure S2. EWSR1 canine retrogene: multiple protein sequence alignment of dog retrogene and the parent EWSR1 gene from diverse representative vertebrates. Top sequences are from EWSR1 proteins across phylogeny. Bottom sequences are of related proteins in humans (see [1]). Three carnivores mentioned in the main article are also included. Alignment was performed using Clustal Omega (EMBL-EBI [2]). Black highlighting represents consensus sites, conservative amino acid differences are grey, and non-consensus sites in EWSR1 canine retrogene are yellow. Protein domains were determined using the ancestral EWSR1 protein from dog and the NCBI Domain Search algorithm; and are shown as a single letter abbreviation in blue highlighting below the alignment: Atrophin-1 super family (p=1.4E-4); zf-RanBP (p=5.3E-10); and RRM_EWS (p=2.5E-54). The Nuclear Localization Signal motif (aka, PY motif) is shown in pink [2]. The EWSR1 NLS is taken from Zakarayan and Gehring [3], the putative ZRANB2 NLS motif is taken from a prediction by Wang et al. [4], RBM5 NLS is taken from Oh et al. [5], RBM10 NLS1 is taken from Xiao et al. (it is the most C-terminal of 3 experimentally derived NLS's; [6]) and TEX13A NLS is taken from Kwon et al. [7]. Species names and GenBank accession numbers follow: Dog retrogene [Canis lupus familiaris, ENSCAFG00000000749], Dog [Canis lupus familiaris, XP_865186.1], Cat [Felis catus, XP_019670384.1], Panda [Ailuropoda melanoleuca, XP_002915634.1], Ferret [Mustela putorius furo, XP_004766556.1], Human [Homo sapiens, NP_053733.2], Rat [Rattus norvegicus, XP_003749731.1], Cow [Bos Taurus, NP_001103270.1], Koala [Phascolarctos cinereus, XP_020852013.1], Big brown bat [Eptesicus fuscus, XP_008140693.1], Yangtze River dolphin [Lipotes vexillifer, XP_007461224.1], European shrew [Sorex araneus, XP_004615678.1], Elephant [Loxodonta Africana, XP_010597062.1], Armadillo [Dasypus novemcinctus, XP_023445520.1], Platypus [Ornithorhynchus anatinus, XP_001516464.2], Chicken [Gallus gallus, XP_015150340.1], Frog [Xenopus tropicalis, NP_989309.1], Zebrafish [Danio rerio, NP_001108610.1].

References

1. Nguyen CD, Mansfield RE, Leung W, Vaz PM, Loughlin FE, Grant RP, Mackay JP: **Characterization of a family of RanBP2-type zinc fingers that can recognize single-stranded RNA.** *J Mol Biol* 2011, **407:**273-283.

2. Sievers F, Wilm A, Dineen D, Gibson TJ, Karplus K, Li W, Lopez R, McWilliam H, Remmert M, Soding J, et al: **Fast, scalable generation of high-quality protein multiple sequence alignments using Clustal Omega.** *Mol Syst Biol* 2011, **7:**539.
3. Zakaryan RP, Gehring H: **Identification and characterization of the nuclear localization/retention signal in the EWS proto-oncoprotein.** *J Mol Biol* 2006, **363:**27-38.
4. Wang X, Du X, Li H, Zhang S: **Identification of the Zinc Finger Protein ZRANB2 as a Novel Maternal Lipopolysaccharide-binding Protein That Protects Embryos of Zebrafish against Gram-negative Bacterial Infections.** *J Biol Chem* 2016, **291:**4019-4034.
5. Oh JJ, West AR, Fishbein MC, Slamon DJ: **A candidate tumor suppressor gene, H37, from the human lung cancer tumor suppressor locus 3p21.3.** *Cancer Res* 2002, **62:**3207-3213.
6. Xiao SJ, Wang LY, Kimura M, Kojima H, Kunimoto H, Nishiumi F, Yamamoto N, Nishio K, Fujimoto S, Kato T, et al: **S1-1/RBM10: multiplicity and cooperativity of nuclear localisation domains.** *Biol Cell* 2013, **105:**162-174.
7. Kwon JT, Jin S, Choi H, Kim J, Jeong J, Kim J, Cho C: **TEX13 is a novel male germ cell-specific nuclear protein potentially involved in transcriptional repression.** *FEBS Lett* 2016, **590:**3526-3537.
